# Exception to the Rule: Genomic Characterization of Naturally Occurring Unusual *Vibrio cholerae* Strains with a Single Chromosome

**DOI:** 10.1155/2017/8724304

**Published:** 2017-08-29

**Authors:** Gary Xie, Shannon L. Johnson, Karen W. Davenport, Mathumathi Rajavel, Torsten Waldminghaus, John C. Detter, Patrick S. Chain, Shanmuga Sozhamannan

**Affiliations:** ^1^Los Alamos National Laboratory, Biosciences Division, Genome Science, Los Alamos, NM 87545, USA; ^2^School of Computer, Mathematical and Natural Sciences, Morgan State University, Baltimore, MD 21251, USA; ^3^LOEWE Centre for Synthetic Microbiology-SYNMIKRO, Philipps-Universität Marburg, Hans-Meerwein-Str. 6, 35032 Marburg, Germany; ^4^Tauri Group, LLC, Alexandria, VA 22310, USA; ^5^Defense Biological Product Assurance Office, 110 Thomas Johnson Drive, Frederick, MD 21702, USA

## Abstract

The genetic make-up of most bacteria is encoded in a single chromosome while about 10% have more than one chromosome. Among these, *Vibrio cholerae*, with two chromosomes, has served as a model system to study various aspects of chromosome maintenance, mainly replication, and faithful partitioning of multipartite genomes. Here, we describe the genomic characterization of strains that are an exception to the two chromosome rules: naturally occurring single-chromosome *V. cholerae*. Whole genome sequence analyses of NSCV1 and NSCV2 (natural single-chromosome vibrio) revealed that the Chr1 and Chr2 fusion junctions contain prophages, IS elements, and direct repeats, in addition to large-scale chromosomal rearrangements such as inversions, insertions, and long tandem repeats elsewhere in the chromosome compared to prototypical two chromosome *V. cholerae* genomes. Many of the known cholera virulence factors are absent. The two origins of replication and associated genes are generally intact with synonymous mutations in some genes, as are *recA* and mismatch repair (MMR) genes *dam*, *mutH*, and *mutL*; MutS function is probably impaired in NSCV2. These strains are ideal tools for studying mechanistic aspects of maintenance of chromosomes with multiple origins and other rearrangements and the biological, functional, and evolutionary significance of multipartite genome architecture in general.

## 1. Introduction

The genetic endowment of the majority of bacteria (~90%) is inherited in a single chromosome of their respective genomes [1]. An exception to this rule is the presence of two chromosomes in *V. cholerae*, the causative agent of cholera disease [2, 3]. In fact, all tested genera/species in the family *Vibrionaceae* possess two chromosomes [4]. The two *V. cholerae* chromosomes (Chr1 and Chr2) are of unequal sizes (~2.96 Mb and ~1.07 Mb, resp.). Most of our knowledge on the control of chromosome maintenance of multipartite genomes is derived from studies on *V. cholerae* [5–7]. It was postulated that Chr2 is derived from plasmid based on its plasmid-like origin and evolved into a secondary chromosome by acquiring additional layers of regulation for its replication [3, 8]. Although Chr1 encodes the majority of the housekeeping genes and is considered as the main chromosome, Chr2 also harbors essential genes beside many genes with unknown functions [9–11]. The genes on Chr1 and Chr2 are differentially expressed in specific niches. For example, when the bacterium was grown midexponentially in rabbit ileal loops, it showed expression of many more genes of Chr2 than those expressed in aerobically grown cells in rich medium and harvested at the midexponential phase [12]. Majority of these are probably important niche-specific genes and hence expressed preferentially in ileal loop. The results were similar when the bacteria were collected from stools of cholera patients [13].

Chr1 replication follows the traditional *E. coli* paradigm in that the replication origin, *ori1*, contains multiple DnaA boxes where DnaA binds to initiate DNA replication [14–16]. A *V. cholerae* Chr1 minireplicon can replicate in *E. coli* without the need for *V. cholerae*-transacting factors but merely using *E. coli* proteins such as DnaA. Similarly, *V. cholerae ori1* could functionally substitute *E. coli* replication origin *oriC* [11, 14, 16].

The *V. cholerae ori2* resembles those of low-copy-number plasmids such as P1 and F in that it contains an array of repeats (iterons) where Chr2-specific initiator protein, RctB, binds to unwind the DNA for *ori2* firing [14, 17]. In *V. cholerae*, Chr1 initiates at the onset of the replication period while initiation of Chr2 is delayed and occurs only when 2/3 of the Chr1 replication has already been completed. Because Chr2 is 1/3 the size of Chr1, both chromosomes terminate their replication roughly at the same time [18, 19]. It was found recently that a site, termed *crtS* (Chr2 replication triggering site), present on Chr1 triggers *ori2* initiation when it is replicated [20, 21].

The two chromosomes of *V. cholerae* are longitudinally arranged in the cell [22]. While Chr1 appears to be spread along the entire longitudinal axis of the cell, Chr2 is restricted to the younger half of the cell. In newborn cells, Chr1 extends from the old pole to the new pole and Chr2 extends from midcell to the new pole [22]. The differential positioning of the chromosomes within the cell is accompanied by a distinct segregation choreography owing to chromosome-specific ParAB/parS-based segregation systems. [22–24]. One of the last steps in chromosome segregation before cell division involves the resolution of dimeric chromosomes that are frequently produced by homologous recombination between sister chromatids following DNA damage [25]. In *V. cholerae*, dimers of Chr1 and Chr2, are resolved by the action of the same machinery, XerC and XerD site-specific recombinases at the *dif* sites (*dif1* and *dif2*), located in the *ter* regions of Chr1 and Chr2, respectively [26]. While the *ter* regions of both chromosomes replicate at the same time point within the cell cycle, Chr1 sister termini are held together at midcell much longer than Chr2 sister termini [22]. The MatP/matS system was found to impede separation of Chr1 sister termini and restrict movement of Chr2 sister termini to allow processing by FtsK right before cell division [27]. In addition, a nucleoid-associated, FtsZ-binding protein termed SlmA has been shown to be required for blocking septal ring assembly. SlmA is a DNA-associated division inhibitor that is directly involved in preventing Z ring assembly on portions of the membrane surrounding the nucleoid [28].

Chr2 is indispensable for viability of the cell since elimination of Chr2 is lethal due to multiple “suicidal” toxin-antitoxin systems encoded by Chr2 [29–31]. *V. cholerae* with single chromosomes has been created by genetic engineering to fuse the two natural chromosomes [32]. Chromosomal fusions in *V. cholerae* were also isolated as suppressor mutations for a deletion of the *dam* (DNA adenine methyl transferase) gene [33]. Dam is essential for replication of Chr2 because the initiator protein RctB binds to the target sites in *ori2* only when they are methylated [14, 16, 32]. The only way to replicate Chr2 without a functional *ori2*, that is, unmethylated *ori2*, was a Chr1 and Chr2 chromosomal fusion that permits replication of the entire chromosome from *ori1* [33]. In *dam-*suppressor mutants, Chr1 and Chr2, fusions had occurred either by homologous recombination between IS elements or site-specific recombination between *dif* sites. Interestingly, fusion occurred preferentially in the terminus regions of the chromosomes [33]. Recently, it was found that chromosomal fusion in *V. cholerae* can also occur as a suppressor of Δ*crtS* mutations [20].

Until recently, evidence for a naturally occurring *Vibrio* with fused chromosomes was missing. In an attempt to assess the genomic diversity of non-O1/non-O139 *V. cholerae*, a whole genome mapping strategy was applied on a well-defined, geographically, and temporally diverse strain collection, the Sakazaki serogroup type strains [34]. In that study, the whole genome map data on 91 of the 206 serogroup type strains supported the hypothesis that *V. cholerae* has an unprecedented genetic and genomic structural diversity with very few clonal complexes. Fortuitously, chromosomal fusions in two unusual strains that possess a single chromosome instead of the two chromosomes usually found in *V. cholerae* were discovered which was further confirmed by pulse field gel electrophoresis (PFGE) [35].

Earlier, we reported the whole genome sequencing and generation of a gapless single-contig sequence of the two unusual single-chromosome strains, 1154-74 (serogroup O49) and 10432-62 (serogroup O27), hereafter referred to as NSCV1 and NSCV2, respectively [36]. In the current paper, we report further genome sequence analyses of NSCV1 and NSCV2. We delineate the Chr1 and Chr2 fusion junctions, other structural anomalies such as indels, inversions and duplications, salient features of their gene content and the origins of replication, and their potential activity. Furthermore, we analyze the genes involved in replication of the multiple origins in the same chromosome. Thus, the primary focus of this paper is to lay the foundation for future studies on functional and mechanistic aspects of chromosome and structural maintenance in these unusual *V. cholerae* strains.

## 2. Materials and Methods

### 2.1. Bacterial Strains and Growth Conditions

*V. cholerae* 1154-74 is a strain isolated in India in 1974 from a diarrheal sample, and it belongs to the O49 serogroup. *V. cholerae* 10432-62 is a strain isolated in the Philippines in 1962 from a diarrheal sample, and it belongs to the O27 serogroup. These strains are referred to as NSCV1 and NSCV2, respectively, in this manuscript. The corresponding Los Alamos National Labs (LANL) Sequencing Center designations are VAAO49 and VABO27, and the old locus tags in the Gen Bank entries carry these designations. The bacterial strains were grown in LB medium at 37°C using normal standard laboratory protocols. Genomic DNAs for sequencing were extracted using Qiagen genomic DNA extraction kits (Qiagen Inc.).

### 2.2. Whole Genome Sequencing Method

The draft genomes of *Vibrio cholerae* strains NSCV1 (1154-74) and NSCV2 (10432-62) were generated at the LANL Genome Science Group using a combination of Illumina [37] and 454 technologies [38]. For each genome, we constructed and sequenced an Illumina GAiiX shotgun library, a 454 Titanium standard library and a paired end 454 library, and a Pacific Biosciences RS long read library (P4-C2 chemistry). The 454 Titanium standard data and the 454 paired end data were assembled together with Newbler, version 2.3-PreRelease-6/30/2009. The Newbler consensus sequences were computationally shredded into 2 kb overlapping fake reads (shreds). Illumina sequencing data was assembled with VELVET, version 1.0.13 [39], and the consensus sequence was computationally shredded into 1.5 kb overlapping fake reads (shreds). We integrated the 454 Newbler consensus shreds, the Illumina VELVET consensus shreds, the PacBio subreads, and the read pairs in the 454 paired end library using parallel phrap, version SPS - 4.24 (High Performance Software, LLC). Illumina data were used to correct potential base errors and increase consensus quality using the software Polisher developed at Joint Genome Institute (JGI) [40]. Possible misassemblies were corrected using gapResolution [41], or Dupfinisher [42]. Completed genome assemblies were compared to optical maps to ensure consensus. Each final assembly consisted of a single ca 4.1 Mb chromosome.

### 2.3. Genome Annotation

Assembled genomes were annotated using a modified version of the IGS Annotation Engine (released by the University of Maryland, Institute for Genome Sciences at the School of Medicine) on an Ergatis workflow manager. Annotated genomes are available in GenBank under accession numbers NSCV1-1154-74: NZ_CP010811.1 and NSCV2-10432-62: NZ_CP010812.1. Post assembly genome sequence analysis was done using the CLC Genomic Workbench version 6.5.

Annotation of the assembled genome sequence was also carried out with genome annotation systems at LANL EDGE server https://bioedge.lanl.gov/ [43] and RAST server [44]. A combined gene prediction strategy was applied by means of the GLIMMER 2.0 system and the CRITICA program suite [45] along with post processing by the RBSfinder tool [46]. tRNA genes were identified with tRNAscan-SE [47]. The deduced proteins were functionally characterized by automated searches in public databases, including SWISS-PROT and TrEMBL [48], Pfam [49], TIGRFAM [50], InterPro [51], and KEGG [52]. Each gene was functionally classified by assigning clusters of orthologous group (COG) number and corresponding COG category [53].

### 2.4. Genomic Comparison

Comparative genome analyses of NSCV1 and NSCV2 to other *V. cholerae* strains MS6, O1 biovar El Tor str. N16961, and O139 MO10 were performed by using a set of tools available at LANL and RAST servers. Homology searches were conducted at the nucleotide and amino acid sequence level using BLAST [54]. To obtain a list of orthologs from bacteroidetes genomes, a perl script that determines bidirectional best hits was written. For example, genes g and h are considered orthologs if h is the best BLASTP hit for g and vice versa. *E* values of 10^−15^ were acceptable. A gene is considered strain specific if it has no hits with an *E* value of 10^−5^ or less. The genome comparisons at the nucleotide level were carried out with genome alignment tools, such as MUMmer2 [55], NUCmer [56], the Artemis Comparison Tool (ACT) [57], and WebAct (http://www.webact.org/WebACT/home) at Imperial College, London.

The MUMmer package [58] programs nucmer, repeat_match, and exact_tandems were used for analysis of repeat regions. To identify long inexact genomic repeats, the nucmer program was used with the options –maxmatch and –nosimplify. The resulting dotplot was obtained using mummerplot. The repeat_match and exact_tandems programs were run with default arguments. Tandem repeats finder [59] and inverted repeats finder [60] were run with recommended default arguments to identify tandem and inverted repeats, respectively. Phage islands and putative genomic islands were identified using PHAST (PHAge Search Tool) [61] and Islandviewer [62], respectively. The circular genome diagram (Figure S3 available online at https://doi.org/10.1155/2017/8724304) was drawn using the DNAplotter [63]. Blast Ring Image Generator (BRIG) software was used to generate genome comparison views presented in Figure S2 [64]. Multiple genome alignment shown in Figure S1 was created using progressive MAUVE [65].

## 3. Results and Discussion

### 3.1. Whole Genome Sequencing of *V. cholerae* Strains NSCV1-(1154-74/VAA_O49 and NSCV2-(10432-62/VAB_O27)

**Figure 1:**
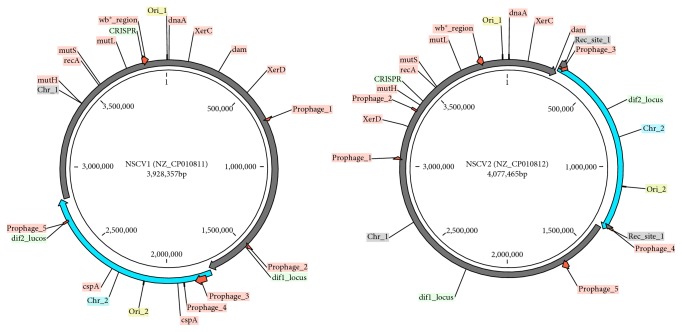
Circular genome maps of NSCV1 (1154-74_VAAO49) and NSCV2 (10432-62_VABO27). Fusion of Chr1 (dark grey) to Chr2 (blue) is shown in the circle at the respective locations. Various unique features such as prophages and the origins of replication- and replication-associated genes are indicated around the circles.

Whole genome mapping (WGM) also known as optical mapping and PFGE data provided evidence of Chr1 and Chr2 fusions in *V. cholerae* strains NSCV1 (1154-74) and NSCV2 (10432-62) [35]. We performed whole genome sequencing of the two *V. cholerae* strains in order to understand the *molecular mechanisms* that might have led to Chr1 and Chr2 fusions in these strains [36]. We generated Roche 454 (21× and 29×), Illumina (2178× and 507×), and Pac Bio RS (350× and 190×) sequence data at the indicated coverages for NSCV1 and NSCV2, respectively, and used a hybrid assembly approach that resulted in a single-contig, gapless genome (Table S1). Further resolution of the ambiguous regions was carried out using Sanger-based primer walking according to Las Alamos National Labs (LANL) genome finishing pipeline yielding a final, finished, and closed single-contig sequence [66]. Consistent with the WGM results and in contrast to the previous genomic reports of *V. cholerae* and its close neighbors [2–4], these two sequences assembled into genomes consisting of a single chromosome of approximately the same size as the two expected chromosomes added together. All other genomic statistics for NSCV1 and NSCV2 appear to be highly similar to other *V. cholerae* genomes currently available in GenBank (Table S1). The genomes of NSCV1 and NSCV2 are comprised of a single circular chromosome of 3,928,357 and 4,074,462 bp in length with an overall G + C content of 47.73% and 47.66%, respectively (Figure 1). Following the sequencing convention, the nucleotide position 1 was placed upstream of the *dnaA* gene (VAA_049_1 and VAB_027_1) encoding the chromosomal replication initiator protein. Chr2 portion of NSCV1 and NSCV2 spans from genome positions 1,720,246–2,772,395 (1,052,150 bps) and 297,221–1,375,947 (1,078,727 bps), respectively. All other NSCV1 and NSCV2 genome statistics are presented in Table S1.

### 3.2. Whole Genome Sequence Comparison of NSCV1 and NSCV2 to Other *V. cholerae*

DNA sequence of *V. cholerae* strain MS6 is the closest match to NSCV1 and NSCV2 chromosomes in public genome databases based on whole genome sequence homology [67]. MS6 is a *V. cholerae* O1 strain with a novel genetic background designated in Thailand–Myanmar and isolated from a stool sample of a diarrheal patient [68]. NSCV1 and NSCV2 genome sequences exhibit a high degree of synteny with MS6, N16961, MO10, and TSY216 as visualized in the Mauve alignment despite genetic rearrangements such as insertions and inversions across the chromosome (Figure S1) [69]. A pairwise Megablast comparison of NSCV1 and NSCV2 genomes to four pathogenic *V. cholerae* (N16961, MO10, MS6, and TSY 216) was performed. At 85%, identity > 88–94% is shared between NSCV1 and NSCV2 compared to N16961, MS6, or MO10 genomes and 74% and 80% is shared between NSCV1 and NSCV2 compared to TSY216, respectively. The unique regions varied from 5 to 10% (Table S2).

To further delineate the differences between NSCV1 and NSCV2 against all other genomes, the locations and gene content of the unique regions were determined (Table S3) and displayed as BRIG view (Figure S2). In the whole genome comparison of NSCV1 and NSCV2 to *V. cholerae* strains MS6, N16961, and TSY216, large regions (~10 kb or more) missing in comparator strains are highlighted in the outer circle of the BRIG view (Figure S2). As expected, many features (e.g., prophages) that are unique to NSCV1 and NSCV2 are absent in the pathogenic strains and similarly, many of the known virulence regions (e.g., CTX and VPI) that are present in pathogenic *V. cholerae* such as N16961 are absent in NSCV1 and NSCV2. In Chr2 segment, the super integron region is present in NSCV1 and NSCV2 with many subtle indels/variations.

### 3.3. Comparison of Genomic Content of NSCV1 and NSCV2 to Pathogenic *V. cholerae*

**Figure 2:**
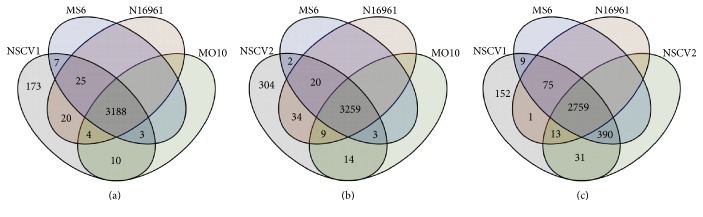
Comparison of the genomic content of NSCV1 (a), NSCV2 (b), and NSCV1 and NSCV2 (c) to various other genomes. Venn diagram showing the number of NSCV1 or NSCV2 predicted CDS with significant homology (1e^−5^) with the predicted products of the near neighbors: *V. cholerae* MS6, N16961 (serogroup O1 biovar El Tor), and O139 MO10 (serogroup O139). The number outside the circles (173 or 304) represents the number of NSCV1 or NSCV2 CDS that does not have significant homologs in the three strains compared. Conserved genes among them were defined by whole-genome pairwise sequence comparisons using the sequence-based comparison tool in RAST.

Further analysis of the genomic content of NSCV1 and NSCV2 was performed by comparing the genome annotations. We were interested to see if there are any differences in the genetic content that might explain the mechanism of genomic fusion. Overall, the number of CDS encoded by NSCV1 and NSCV2 is very similar to other *V. cholerae* (Table S1). NSCV1 and NSCV2 have 2759 CDS in common with all other genomes compared here. Comparison of the genomes of NSCV1 and NSCV2 with MS6, N16961, and MO10 (an epidemic *V. cholerae* strain belonging to the O139 serogroup) revealed that the four organisms have approximately 3188/3259 genes in common, depending on whether NSCV1 or NSCV2 is used as the query, respectively (Figure 2). There are 173/304 CDSs unique to NSCV1 and NSCV2, respectively.

### 3.4. Genomic Islands, Prophages, and Other Mobile Genetic Elements

In the NSCV1 and NSCV2 chromosomes, 49 and 20 genomic islands amounting to 677, 625 bps and 205, 247 bps or 16.32% and 4.94% of the entire genome, respectively, were detected. Clustered regularly, interspaced short palindromic repeat (CRISPR) element is a widely found defense mechanism of prokaryotes against entry of foreign DNA including plasmids and phages [70]. In the NSCV1 and NSCV2 chromosomes, one CRISPR element was located at 3,606,088–3,606,203 bps and 3,572,464–3,572,579 bps in NSCV1 and NSCV2 genomes, respectively. The NSCV1 and NSCV2 genomes contained 58 and 55 mobile genetic elements, respectively, that encode phage integrases, transposases, and site-specific recombinase (Figure S3), compared to 46 mobile element genes in *V. cholerae* MS6. NSCV1 has 5 prophages located at (1) 685,729–702,622; (2) 1,458,739–1,476,484; (3) 1,756,187–1,818,403; (4) 1,879,405–1,883,642; and (5) 2,643,793–2,650,802. Prophages 3 and 5 are at the immediate boundary region and inside of the Chr2 insertion locus. Only prophage 3 at the 5′ end of Chr2 insertion appears to be intact and encodes 56 CDS. NSCV2 has 5 prophages located at (1) 3,113,341–3,130,219; (2) 3,416,918–3,428,588; (3) 299,327–349,041; (4) 1,359,430–1,369,981; and (5) 1,676,903 to 1,714,350. Only prophage 5 is intact whereas the other four are defective or incomplete. Prophages 3 and 4 are at the immediate flanking region of the Chr2 insertion junction (Figure 1).

### 3.5. Virulence and Antimicrobial Resistance Genes

*V. cholerae* strains NSCV1 and NSCV2 were isolated from patients exhibiting atypical cholera symptoms [34]. It is of interest to see if these strains carry any of the usual *V. cholerae* virulence factors and if not, what other virulence factors or toxins might be present that would explain the diarrheal symptoms. The major virulence factor of *V. cholerae*, the cholera toxin encoded by *ctxAB* genes, is not found in NSCV1; however, other toxin genes *ace* and *zot* and RTX toxin cluster can be found, in addition to *toxRS* genes. The toxin genes are absent in NSCV2. NSCV1 and NSCV2 each has 57 genes encoding putative resistance to antibiotics and toxic compounds such as colicin V, bacteriocin, cobalt-zinc-cadmium, copper homeostasis, fluoroquinolones, and multidrug resistance efflux pumps.

### 3.6. O-Antigen Regions

**Figure 3:**
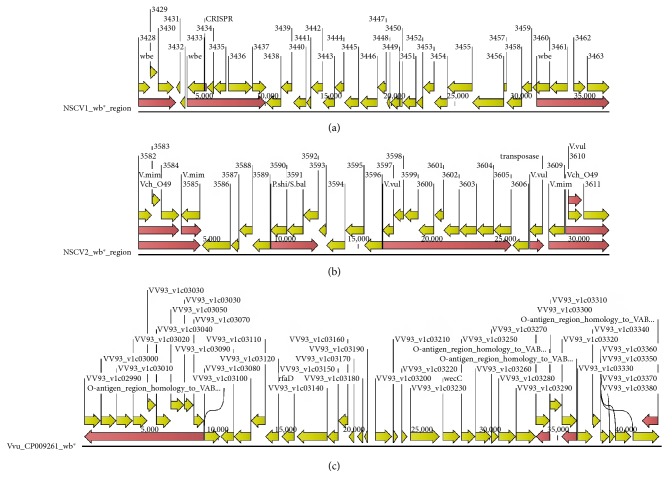
Genetic maps of O-antigen regions (*wb*^∗^) of NSCV1 and NSCV2. The various genes and their orientations are indicated by the arrows. Homologous regions to other serogroups are indicated by the red arrows. The *V. vulnificus wb*^∗^ cluster that has extensive homology to NSCV2 *wb*^∗^ cluster is shown in (c).

NSCV1 and NSCV2 belong to serogroups O49 and O27, respectively, and as seen in other serogroups of *V. cholerae*, the O-antigen biosynthesis genes are encoded in a cluster (*wb*^∗^) on Chr1 part of the respective genomes from 3,777,887–3,815,081 (CDS VAAO49_3428-VAAO49_3463) and 3,888,708–3,920,841 (CDS VABO27_3582-VABO27_3611) of NSCV1 and NSCV2, respectively. Also, as seen in other *wb*^∗^ regions, the boundary regions of this cluster are highly conserved whereas the region between the conserved genes is highly divergent. More specifically, in the NSCV1 *wb*^∗^ region, 2 segments of 2974 bps and 6212 bps at the left end and another segment of 5733 bps at the right end, respectively, are 96% and 94% identical to those in the *wbe* region (O1 serogroup). A blast analysis of the *wb*^∗^ region of NSCV2 revealed segments that are similar to *V. vulnificus* (GenBank Accession # CP009261) gene cluster (around 42 kb) with 4 segments that are >65–85% identical, ranging in length from ~1.0 kb to 8.831 kb to the NSCV2 *wb*^∗^ cluster. In addition, identities to *V. mimicus* at 83–92% in segments of 4176 bps, 2789 bps, and 1410 bps and to *Plesiomonas shigelloides* and *Shewanella baltica* at ~80% (3281 bps), and to NSCV1 at 94–96% (4325, and 3007 bps) identities were also found as depicted in Figure 3.

### 3.7. Identification of Large Tandem Repeats and Inversion

A closer analysis of the WGMs (whole genome maps) of NSCV1 and NSCV2 strains revealed a large duplication in the general region of 1240 and 1506 kb on a reference genome (M66–2) coordinates based on optical restriction maps [35]. Experimental WGM data is further supported by in silico WGM generated from whole genome sequence data of NSCV1 and NSCV2 (Figure S4). Sequence read mapping results showed that there is a large duplication of 200 kbs and 70 kbs regions in NSCV1 and NSCV2, respectively, as evidenced by >1×, but <2×, depth of average coverage of the genome at 1,503,484 to 1,718,861 bps in NSCV1 and 2,221,105 to 2,300,924 bps in NSCV2 compared to that in the rest of the genome (Figure S5). Manual validation indicated that the 200 kbs and 70 kbs regions indeed are fragmental duplications, existing among subpopulation of NSCV1 and NSCV2 cells, respectively. Both 1 copy and 2 copy versions of genome assembly have enough evidence supporting variations among population. These duplicated sequences spanned from positions 1,503,484 to 1,718,861 (unit 1) and from 1,718,862 to 1,934,269 bp (unit 2) in NSCV1 and from positions 2,221,105 to 2,300,924 (unit 1) and from 2,300,925 to 2,380,745 (unit 2) in NSCV2 in genome sequences with repeats included (Figure S6). The duplicated sequences span a total length of 430,785 bp and 159,640 bp on Chr1 region and encode 177 and 59 duplicated genes in NSCV1 and NSCV2, respectively. It should be noted that the two copies of repeat units are not identical. There is a 31 bp indel between the two copies in NSCV1, which resides at the locus of VAA_049_1509, encoding exonuclease family protein. There are 5 single bp indels between the two copies in NSCV2. Although tandem repeats are present at different locations on NSCV1 and NSCV2, there is a 40 kb DNA segment that overlapped at the 5′ end of NSCV1 and NSCV2 repeats with 98% identities (Figure S7). However, the tandem duplications could not be verified by PCR of the junctions since the orientation of the two copies cannot be ascertained in the assembly. Hence, we have decided to keep the genome sequence with a single copy as the ref seq (GenBank submission) for all the analyses. This long duplication anomaly could not be unequivocally resolved with the existing sequence data. In addition, there is a large inversion from 1,477,189 to 3,484,983 bps in NSCV2 compared to MS6 chromosome 1 region from 612,067 to 2,541,958 bps (Figure S1b).

### 3.8. Mapping the Fusion Junctions and Possible Mechanism of Genome Fusion

Chromosomal fusions were observed previously in suppressor mutants resulting from a genetic screen [33]. These fusions were analyzed in detail and found to occur via two different mechanisms, either by homologous recombination at IS elements or by site-specific recombination at *dif* sites [32] or as transient chromosomal fusions in *ΔcrtS* suppressor mutants [20]. In this study, we analyzed the fusion sites of NSCV1 and NSCV2 in detail to see if any one of these mechanisms also led to chromosomal fusion in the natural single-chromosome isolates. The circular single chromosomes of NSCV1 and NSCV2 are shown in Figure 1, and along with N16961, Chr1- and Chr2-concatenated circular maps are shown in Figure S2, and the genome sequence statistics are provided in Table S1. The insertion sites on Chr1 backbone (as an accepter) and Chr2 backbone (as a donor) are not the same for NSCV1 and NSCV2 (see below). Artemis Comparison Tool views (zoomed in view) of the fusion junctions are presented in Figures S8 and S9. Further description of the hypothetical events leading to the fusions is provided below.

### 3.9. Chr1 and Chr2 Fusion Junctions in *V. cholerae* Strain NSCV1

**Figure 4:**
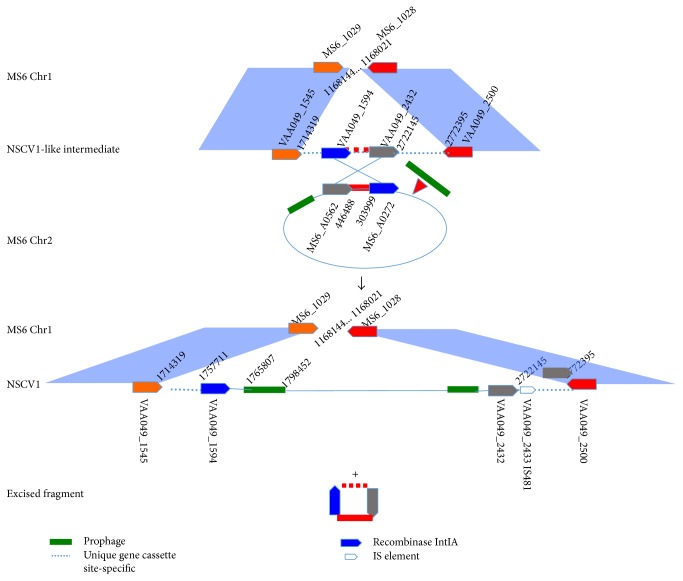
Putative recombination event that resulted in Chr1 and Chr2 fusions in NSCV1. Top panel: MS6 Chr1 CDS at the fusion junction and the same location in a putative NSCV1-like intermediate and the MS6 Chr2 circle and the codons at the fusion junction are depicted. The cross-over region between Chr1 and Chr2 is indicated by the long X. A prophage insertion event to the right fusion junction prior or post to Chr 2 fusion is depicted by a green line. Bottom panel: The recombination products after fusion event are shown with NSCV 1 in the middle and an excision product that contains one copy of the cross-over genes with the intervening sequences at the bottom.

A closer examination of the Chr1-Chr2 insertion junction in NSCV1 to Chr1 and Chr2 sequences of MS6 indicates that multiple events probably occurred before the final NSCV1 was derived. In an NSCV1-like intermediate, on Chr1 between VAA049_1545 and VAA049_2500, there probably was an insertion of MS6_A0562- and MS6_A0272-like CDS (Chr2 CDS) (Figure 4() (top panel)). On Chr2, there was an insertion of a prophage to the right of MS6_A0272 (Figure 4() (top panel)). In the next step, Chr2 with the new prophage was inserted into Chr1 via the homologies between the two CDS (VAA049_1594 versus MS6_A0272 and VAA049_2432 versus MS6_A0562), and upon the resolution of the cointegrate, one copy each of the two homologous CDS along with intervening sequences was deleted (Figure 4() (bottom panel)). Thus, Chr2 in NSCV1 spans from 1,714,319 to 2,772,395 bps (including its flanking unique region, Figure 4() (bottom panel)) or from 1,757,711 to 2,722,145 bps (including boundary of Chr2 homologous region) (Figure 4() (bottom panel)) spanning NSCV1 CDS VAAO49_1594–2432. This recombination event likely occurred between MS6_1029 and MS6_1028 or between 1,168,144 and 1,168,021 bp in MS6-like Chr1 background and inserted from the breakpoint between MS6_A0272 and MS6_A0562 or between 303,999 and 446,488 bp on MS6-like Chr2 background (Figure 4() (bottom panel)). There are 2 prophages (3 and 5) closer to the boundaries of Chr2 insertion locus. Flanking the Chr2 insertion sites, there are unique regions of 37,425 bps and 50,250 bps in length at 5′ end and 3′ end, respectively. The role of any of these unique regions in the recombination event or the precise mechanism of recombination cannot be ascertained with available whole genome sequence data in public databases.

### 3.10. Chr1 and Chr2 Fusion Junctions in *V. cholerae* Strain NSCV2

**Figure 5:**
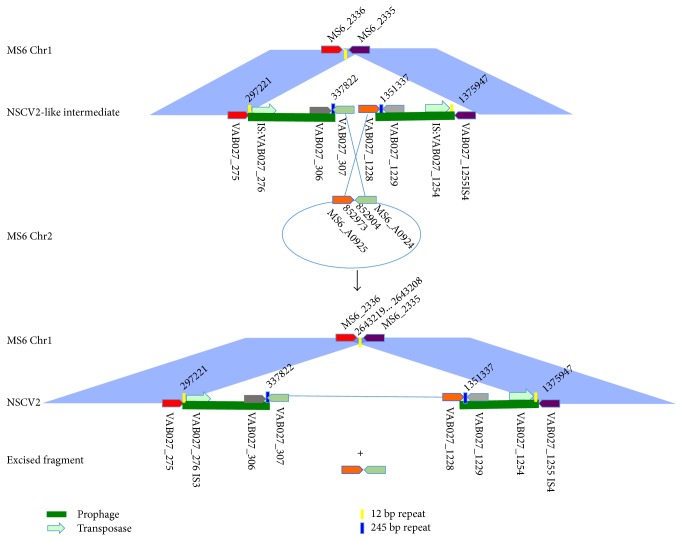
Putative recombination event that resulted in Chr1 and Chr2 fusions in NSCV2. Top panel: MS6 Chr1 CDS at the fusion junction and the same location in a putative NSCV2-like intermediate and the MS6 Chr2 circle and the codons at the fusion junction are depicted. The cross-over region between Chr1 and Chr2 is indicated by the long X. Bottom panel: The recombination products after fusion event are shown with NSCV2 in the middle and an excision product that contains one copy of the cross-over genes with the intervening sequences at the bottom.

A closer examination of the insertion junctions indicates that an NSCV2-like intermediate probably possessed MS6_A0925- and MS6_A0924-like Chr2 CDS on Chr1 flanked by 2 prophages (Figure 5 (top panel)). In that intermediate strain, Chr2 was inserted via recombination of the homologous segments (VAB027_307 versus MS6_A0924 and VAB027_1228 versus MS6_A0925). As a result, Chr2 spans from 337,822 bps to 1,351,337 bps (without its flanking prophage regions between CDS VABO27_307-VABO27_1228) or from 297,221 bps to 1,375,947 bps (including prophage regions between CDS VABO27_275-VABO27_1225). This recombination event likely occurred between 2,643,219 and 2,643,208 bp or between MS6_2336 and MS6_2335 on MS6 Chr1 backbone and inserted between MS6_A0924 and MS6_A0925 break points (852,904–852,973) on MS6 Chr2 backbone (Figure 5 (bottom panel)). The insertion boundary is flanked by 245 bp repeats located at 337,552 to 337,796 bp and 1,351,093 to 1,351,337 bps with 96% identities, then flanked by 2 prophages. In the flanking regions of 2 prophages, there are 12 bp identical repeats (caccgcagggtg) (297,210–297,221 bps and 1,375,947–1,375,958 bps). The 245 bps repeat also overlaps 155 bps with VABO27_1228 that encodes L-threonine 3-dehydrogenase.

As mentioned above for NSCV1, the role of any of these unique regions in the recombination event or the mechanism of recombination that resulted in chromosomal fusion cannot be ascertained with available *V. cholerae* whole genome sequence data. Since the immediate predecessor or recombination intermediate strains are not known, it is difficult to decipher the exact events that led to the NSCV1 and NSCV2. For example, the exact mechanism of recombination (site specific versus generalized recombination) or the precise recombination cross-over points cannot be predicted. It also appears that there is more than one event before the final NSCV1 and NSCV2 arose. The presence of prophages at the insertion junctions leads us to speculate that the Chr2 insertion events in NSCV1 and NSCV2 appear to be mediated by generalized homologous recombination via homologies provided by prophages or parts/genes homologous to prophage genes and mobile gene elements present in both Chr1 and Chr2 in the precursor strain.

### 3.11. Sequence Analysis of the Origins of Replication and Associated Genes

The *ori1* and *ori2* origins of replications in NSCV1 and NSCV2 chromosomes were identified by a homology to the Chr1 and Chr2 origins of MS6. The *ori1* is colocalized with the genes (*rpmH*, *dnaA*, *dnaN*, *recF*, and *gyrA*) often found near the *oriC* in prokaryotic genomes, and origin of the location corresponded with GC nucleotide skew (G − C/G + C) analysis as illustrated in Figure S2 and Figure S3. Based on these data, we assigned base-pair 1 in an intergenic region located in the putative *ori1*. Similarly, *ori2* was located based on sequence homology to the *ori2* in other prototypical *V. cholerae* genomes. The genome positions of the *ori*, their orientation, and genes within the *ori* are indicated in Table 1. A list of putative *dif* sites (chromosome dimer resolution sites) and their locations are provided in Table S4. A previous study had found chromosome fusions in *V. cholerae* as suppressors of impaired Chr2 replication [33]. As mentioned above, some of these fusions had occurred by site-specific recombination of the two *dif* sites of Chr1 and Chr2, respectively. The positions of the intact *dif* sites on the fused chromosomes in NSCV1 and NSCV2 support the notion that this was not the mechanism how fusion occurred in these strains (Table S4). It remains to be seen which of the two *dif* sites on NSCV1 and NSCV2 chromosomes is active and used for chromosome dimer resolution. This might depend on the molecular mechanism involved in positioning the respective XerCD recombinase at the *dif* site.

**Figure 6:**
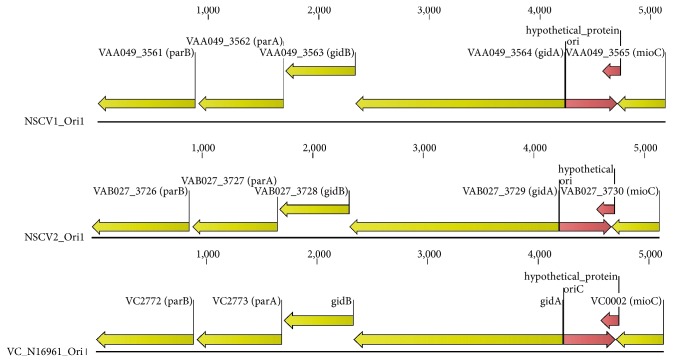
Genetic organization of *ori1* of NSCV1 and NSCV2 in comparison to the respective *ori* in N16961. The old locus tags with known gene designations have been used to indicate the ORFs. The physical *ori* and unannotated ORFs are indicated by red arrows.

**Figure 7:**
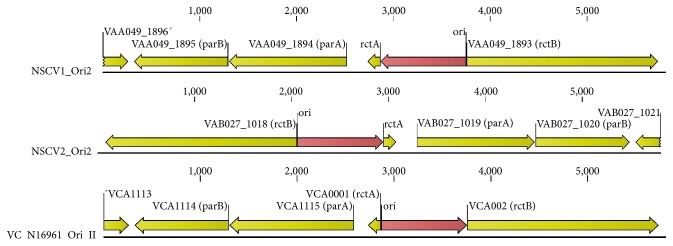
Genetic organization of *ori2* of NSCV1 and NSCV2 in comparison to the respective *ori* in N16961. The old locus tags with known gene designations have been used to indicate the ORFs. The physical *ori* is indicated by a red arrow.

There is extensive genetic conservation in the *ori1* and *ori2* of NSCV1 (Figure 6) and NSCV2 (Figure 7) compared to a prototypical reference genome such as N16961. A closer look at the sequences of the origin regions including the genes within the origin of replication indicated that there is no significant indels between the respective origins compared to a reference genome such as N16961. However, a number of SNPs were found in the origins of replication. The nucleotide and amino acid changes at the origins of NSCV1 and NSCV2 are presented in Table S5. It remains to be analyzed experimentally if both origins on the fused chromosomes are active in replication.

### 3.12. Sequence Analyses of Replication Associated and Mismatch Repair Genes That May Be Potentially Involved in Single-Chromosome Maintenance

Earlier studies have indicated that the *dam* gene is essential for the viability of *V. cholerae*, and depletion of DNA adenine methyl transferase (Dam protein) leads to successful spontaneous fusion of the two chromosomes [33]. Hence, we examined the genetic status of DNA adenine methylase gene (*dam*) in natural single-chromosome *V. cholerae*, NSCV1, and NSCV2 and found the *dam* gene to be intact. We also inspected the status of RecA and the MMR genes since they have been implicated in maintenance of chromosomal rearrangements such as large tandem repeats, inversion, and fusion. The nucleotide and amino acid changes in various replication and MMR genes in NSCV1 and NSCV2 are presented in Table S6. RecA-mediated homologous recombination was probably involved in the fusion event, and the fact that the resolution of the single chromosome into 2 chromosomes has not been observed during normal growth conditions indicates that the RecA in NSCV1 and NSCV2 is nonfunctional or altered due to the presence of multiple SNPs, of which one leads to single-amino acid change in RecA protein (Y305C). Preliminary data indicate that NSCV1 and NSCV2 are probably recombination deficient since construction of recombinants via natural transformation has been unsuccessful. The MMR genes are generally intact except for *mutS*. The *mutS* gene is impaired, that is, deletion of amino acid residues 132–150, in NSCV2. Among the other mismatch repair system genes, *mutH* and *mutL* have single-nucleotide polymorphisms that lead to amino acid changes in the respective proteins. Other replication-associated proteins such as DnaA, ParAB, and XerC have amino acid changes whereas XerD is intact (Table S6). Preliminary data indicate that the *ori1* is active in both strains suggesting a functional DnaA protein. However, the effect of these protein alterations in ParAB, XerC, RecA, and MMR proteins on recombination, replication, and maintenance of chromosomal fusions and other large-scale genome rearrangements described above awaits more stringent functional studies including cloning and intra-/interspecific complementation and these studies are underway.

## 4. Conclusions

All species of the genus Vibrio known to date harbor two chromosomes. Here, we present an exception to this rule by describing the genomic architecture of two natural *V. cholerae* isolates with one fused chromosome. For many years, the quest to understand why and how Vibrios evolved their bipartite genomes remains enigmatic. The strains described here appeared to have taken an evolutionary path backwards and might be instrumental in future unraveling of long-standing questions on chromosome biology in Vibrios. One fascinating question to address is whether the replication of the fused chromosome is dominated by one of the subchromosomes or if they share the two replicons. If the latter is true then this would be the first example of a bacterial chromosome with two active replication origins. A tantalizing idea that has been proposed is that Chr2 is actually a “parasitic replicon.” The concept of selfish replication origins has been established recently [71]. It could be that the *V. cholerae* strains described here are the result of a selfish replicon taking over the “host.” Currently, studies are underway to begin to address these questions to unravel the mystery of a single chromosome with multiple replication origins. A second question that is worth exploring pertains to how the chromosomal fusions are maintained and if and how or under what conditions they ever revert into two separate chromosomes. Many of the genes involved in these functions have suffered changes. It remains to be seen if NSCV1 and NSCV2 strains are defective or functionally altered in any of these genes in order to maintain the chromosomal fusions and other structural alterations.

## Supplementary Material

Figure S1. Mauve alignment of NSCV1 (S1a) and NSCV2 (S1b) with other genomes. NSCV1 and NSCV2 whole genome sequences were aligned to the genome sequences of *V. cholerae* strains MS6, N16961, MO10 and TSY216. The various collinear regions are indicated by different colored blocks. The Chr2 sections of NSCV1 and NSCV2 are indicated by a red line below the scale (bps). The large chromosomal inversion in NSCV2 (Figure S1b) can be seen as the blocks that are indicated on the opposite strand in MS6. Figure S2. BRIG view of genomic comparisons of NSCV1, NSCV2, N16961, TSY216 and MS6. For genome comparisons, default blastn parameters were used. Unique regions (Uni_region) ˜ 10 kb or more (along with annotation of the region if known) including prophages are indicated around the circle. In Figure 2c, using N16961 as the reference genome, Chr1 and Chr2 sequences are concatenated end to end and the various known virulence markers are indicated. N16961 is a prototypical *V. cholerae* with two chromosomes and the sequences are concatenated here for illustrative purpose only. Figure S3. Circular maps of NSCV1 (S3a) and NSCV2 (S3b) showing various features. Circular map of the NSCV1 (S3a) and NSCV2 (S3b) genomes, showing the distribution of coding sequences, mobile elements, GC content and GC skew. For this analyses the sequences with large tandem repeats were included. From outside to the center: Circles 1 and 2: forward and reverse strand genes; Circle 3: unique genes in NSCV1 or NSCV2 in comparison with *V. cholerae* MS6, serogroup O1 biovar El Tor str. N16961, and serogroup O139 MO10; Circle 4: Chr2 in grey color and large tandem repeat in pink; Circle 5: OriC for Chr1 and 2; Circle 6: Prophage predicted by Phast; Circle 7: Genomic island Predicted by multiple methods: IslandPick, SIGI-HMM and IslandPath-DIMOB; Circle 8: GC content; Circle 9: GC skew. Figure S4. WGM maps compared to in silico generated restriction maps of WGS. Whole genome optical maps (*Afl*II) of NSCV1 (top panel) NSCV 2 (bottom panel) aligned to in silico restriction maps (*Afl*II) using genome assemblies. The region of the sequence that appear as tandem repeats in the optical map are highlighted by the yellow arrows. Figure S5. Genome coverage at tandem repeats of NSCV1 and NSCV2. Sequence reads of NSCV1 and NSCV 2 generated from Illumina and 454 sequencers were mapped against the respective final assemblies and displayed as function of fold coverage vs genome position in the assembly. Note that increased coverage indicated by the star is readily apparent except in the case of 454 SE read data for NSCV1. This anomaly could not be resolved at this time. Figure S6. Genome maps of NSCV1 and NSCV2 with the large tandem repeats. Circular genome maps of NSCV1 and NSCV2 similar to Figure 1 with the tandem repeats included (red features). Fusion of Chr1 (dark grey) to Chr2 (blue) is shown in the circle at the respective locations. Various unique features such as tandem repeats, prophages and the origins of replication and replication associated genes are indicated around the circles. Figure S7. Overlap of repeat regions between NSCV1 and NSCV2. The identity plot indicates the first 40,398 bps of NSCV1 tandem repeat region (total length of the repeat: 196,770 bps) which is 97% identical to the terminal 40,285 bps of NSCV2 tandem repeat region (total length of the repeat: 79,821 bps). Figure S8. Artemis Comparison Tool (ACT) view of NSCV1 Chr1 and Chr2 fusion junction. ACT was used to compare the MS6 Chr1 and Chr2 sequences against NSCV1 sequence. Genome sequences were aligned from the predicted *ori1* and visualized in ACT with a cut-off set to blast scores >500. Red and blue bars indicate regions of similarity in the same orientation (blue) and inverted (red). B) Zoom-in region of MS6-like Chr2 inserted in ChrI. All homologous genes at the insertion boundaries are the same color coded and the 2 prophages are indicated by the green bar. Figure S9. Artemis Comparison Tool (ACT) view of NSCV2 Chr1 and Chr2 fusion junction. ACT was used to compare the MS6 Chr1 and Chr2 sequences against NSCV2. Genome sequences were aligned from the predicted OriC and visualized in ACT with a cut-off set to blast scores >500. Red and blue bars indicate regions of similarity in the same orientation (blue) and inverted (red) respectively. B) Zoom-in region of MS6 like Chr2 inserted in ChrI. All homologous genes at insertion boundaries are the same color coded. The green bars indicate the two prophages. The 12 bps and 245 bps repeats are labeled in yellow and blue line, respectively. Table S1. Whole Genome Sequence Statistics of NSCV1 and NSCV2. Table S2. Nucmer based pairwise comparison of genome sequences. Table S3. Unique Regions ˜10 Kb and over in NSCV1, NSCV2 and N16961 in comparison to others genomes. Table S4. List of potential *dif* sites in NSCV1 and NSCV2. Table S5. Summary of nucleotide and amino acid changes in origins of replication genes and corresponding proteins. Table S6. Summary of nucleotide and amino acid changes in replication associated and mismatch repair genes and corresponding proteins.

## Figures and Tables

**Table 1:** Genome locations of origins of replication and replication-associated genes.

*Ori1 and Ori2 and the genes within Ori*																			
Locus ID	Gene/Locus	N16961_O1						Locus ID	1154-74_O49					Locus ID	10432-62_O27				
		Start		End	Size	Strand	Orientation		Start	End	Size	Strand	Orientation		Start	End	Size	Strand	Orientation

Rep_ori_Chr_1		2956820	2961149/1	806	5136		Clockwise	Rep_ori_Chr_1	3916660	3921794	5135		Clockwise	Rep_ori_Chr_1	4039130	4044265	5136		Clockwise
VC2772	*parB*	2956823	2957704		882	Complement		VAA049_RS18130	3916663	3917544	882	Complement		VAB027_RS18795	4039133	4040014	882	Complement	
VC2773	*parA*	2957731	2958504		774	Complement		VAA049_RS18135	3917571	3918344	774	Complement		VAB027_RS18800	4040041	4040814	774	Complement	
VC2774	*gidB*	2958519	2959151		633	Complement		VAA049_RS18140	3918359	3918991	633	Complement		VAB027_RS18805	4040829	4041461	633	Complement	
VC2775	*gidA*	2959151	2961046		1896	Complement		VAA049_RS18145	3918991	3920886	1896	Complement		VAB027_RS18810	4041461	4043356	1896	Complement	
	*Ori_1*	2961047	2961149	371	474			*Ori_1*	3920887	3921359	473			*Ori_I*	4043357	4043830	474		
VC0001	*hypo*	235	402		168	Complement		VAA049_RS18150	3921224	3921390	167	Complement		VAB027_RS18815	4043694	4043861	168	Complement	
VC0002	*mioC*	372	806		435	Complement		VAA049_RS18155	3921360	3921794	435	Complement		VAB027_RS18800	2269384	2269818	435	Complement	
Rep_ori_Chr_2		1069696	1072315/1	3191	5811		Clockwise	Rep_ori_Chr_2	2096979	2102790	5812		Counterclockwise	Rep_ori_Chr_2	1115023	1120832	5810		Counterclockwise
VCA1113		1068927	1069958		1032			VAA049_RS09630	2102528	2103559	1032	Complement		VAB027_RS05240	1120570	1121601	1032	Complement	
VCA1114	*parB*	1070018	1070989		972	Complement		VAA049_RS09625	2101496	2102476	972			VAB027_RS05235	1119539	1120510	972		
VCA1115	*parA*	1070997	1072220		1224	Complement		VAA049_RS09620	2100271	2101488	1218			VAB027_RS05230	1118314	1119531	1218		
VCA0001	*rctA*	112	246		135	Complement		*rctA*	2099924	2100058	135			*rctA*	1117968	1118102	135		
	*Ori_2*	247	1133		887			*Ori_2*	2099037	2099923	887			*Ori_II*	1117081	1117967	887		
VCA0002	*rctB*	1134	3110		1977			VAA049_RS09615	2097060	2099036	1977	Complement		VAB027_RS05225	1115104	1117080	1977	Complement	

*Replication-associated genes*																			
Locus ID	Gene	N16961_O1						Locus ID	1154-74_O49					Locus ID	10432-62_O27				
		Start	End		Size	Orientation			Start	End	Size	Strand			Start	End	Size	Orientation	

VC2626	*dam*	2796912	2797745		834	Complement		VAA049_RS01410	295053	295886	834			VAB027_RS01350	282403	283236	834		
VC0543	*recA*	574696	575760		1065			VAA049_RS016045	3495035	3496099	1065	Complement		VAB027_RS16670	3602,557	3603621	1065	Complement	
VC0668	*mutH*	715847	716512		666	Complement		VAA049_RS015395	3352580	3353245	666			VAB027_RS16030	3461932	3462597	666		
VC0345	*mutL*	367072	369033		1962			VAA049_RS016985	3680484	3682445	1962	Complement		VAB027_RS17585	3784248	3786209	1962	Complement	
VC0535	*mutS*	565832	568420		2589	Complement		VAA049_RS16080	3502248	3504836	2589			VAB027_RS016705	3609897	3612475	2580		
VC0128	*xerC*	122005	122940		936			VAA049_RS00590	114667	115602	936			VAB027_RS00575	114596	115531	936		
VC2419	*xerD*	2593375	2594283		909	Complement		VAA049_RS02440	498541	499449	909			VAB027_RS15355	3316085	3316993	909	Complement	
VC0012	*dnaA*	7397	8815		1419			VAA049_RS00005	38	1441	1404			VAB027_RS00005	38	1441	1404		
	*dif-1*	1564104	1564131		28	Complement		IGR of VAA049_RS06840-VAA049_RS06845	1476590	1476617	28			IGR of VAB027_RS10790-VAB027_RS10795	2301152	2301179	28	Complement	
	*dif-2*	507983	508010		28			VAA049_RS12025	2643791	2643818	28	Complement		IGR of VAB027_RS03005-VAB027_RS03010	664645	664672	28	Complement	

## Abbreviations

NSCV: Natural single-chromosome vibrio
WGM: Whole genome map (optical map)
WGS: Whole genome sequence
PFGE: Pulse field gel electrophoresis
LANL: Los Alamos National Labs
JGI: Joint Genome Institute
BRIG: Blast Ring Image Generator.

## Additional Points

*Availability of Supporting Data*. Whole genome sequences are available in GenBank under accession numbers NSCV1-1154-74: NZ_CP010811.1 and NSCV2-10432-62: NZ_CP010812.1.
