# Supplementary material for: Exception to the Rule: Genomic Characterization of Naturally Occurring Unusual Vibrio cholerae Strains with a Single Chromosome
Source: Int J Genomics. 2017 Aug 29;2017:8724304. doi: 10.1155/2017/8724304 (PMC5603330; doi:10.1155/2017/8724304)
Supplement: Supplementary file 1 — Figure S1. Mauve alignment of NSCV1 (S1a) and NSCV2 (S1b) with other genomes. NSCV1 and NSCV2 whole genome sequences were aligned to the genome sequences of V. cholerae strains MS6, N16961, MO10 and TSY216. The various collinear regions are indicated by different colored blocks. The Chr2 sections of NSCV1 and NSCV2 are indicated by a red line below the scale (bps). The large chromosomal inversion in NSCV2 (Figure S1b) can be seen as the blocks that are indicated on the opposite strand in MS6. Figure S2. BRIG view of genomic comparisons of NSCV1, NSCV2, N16961, TSY216 and MS6. For genome comparisons, default blastn parameters were used. Unique regions (Uni_region) ˜ 10 kb or more (along with annotation of the region if known) including prophages are indicated around the circle. In Figure 2c, using N16961 as the reference genome, Chr1 and Chr2 sequences are concatenated end to end and the various known virulence markers are indicated. N16961 is a prototypical V. cholerae with two chromosomes and the sequences are concatenated here for illustrative purpose only. Figure S3. Circular maps of NSCV1 (S3a) and NSCV2 (S3b) showing various features. Circular map of the NSCV1 (S3a) and NSCV2 (S3b) genomes, showing the distribution of coding sequences, mobile elements, GC content and GC skew. For this analyses the sequences with large tandem repeats were included. From outside to the center: Circles 1 and 2: forward and reverse strand genes; Circle 3: unique genes in NSCV1 or NSCV2 in comparison with V. cholerae MS6, serogroup O1 biovar El Tor str. N16961, and serogroup O139 MO10; Circle 4: Chr2 in grey color and large tandem repeat in pink; Circle 5: OriC for Chr1 and 2; Circle 6: Prophage predicted by Phast; Circle 7: Genomic island Predicted by multiple methods: IslandPick, SIGI-HMM and IslandPath-DIMOB; Circle 8: GC content; Circle 9: GC skew. Figure S4. WGM maps compared to in silico generated restriction maps of WGS. Whole genome optical maps (AflII) of NSCV1 (top panel) [file 8724304.f1.pptx]

## Slide 1
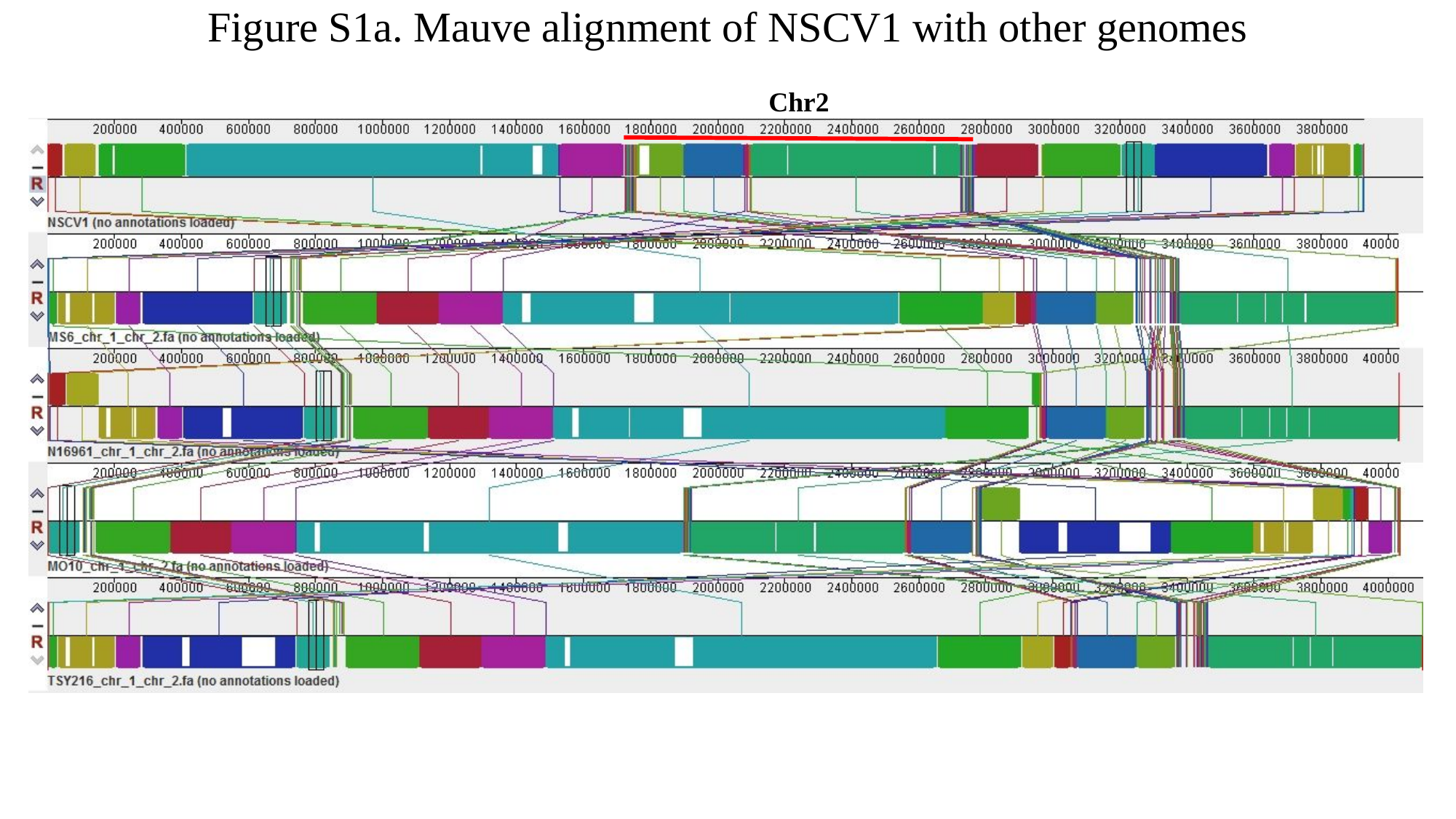

Figure S1a. Mauve alignment of NSCV1 with other genomes
Chr2

## Slide 2
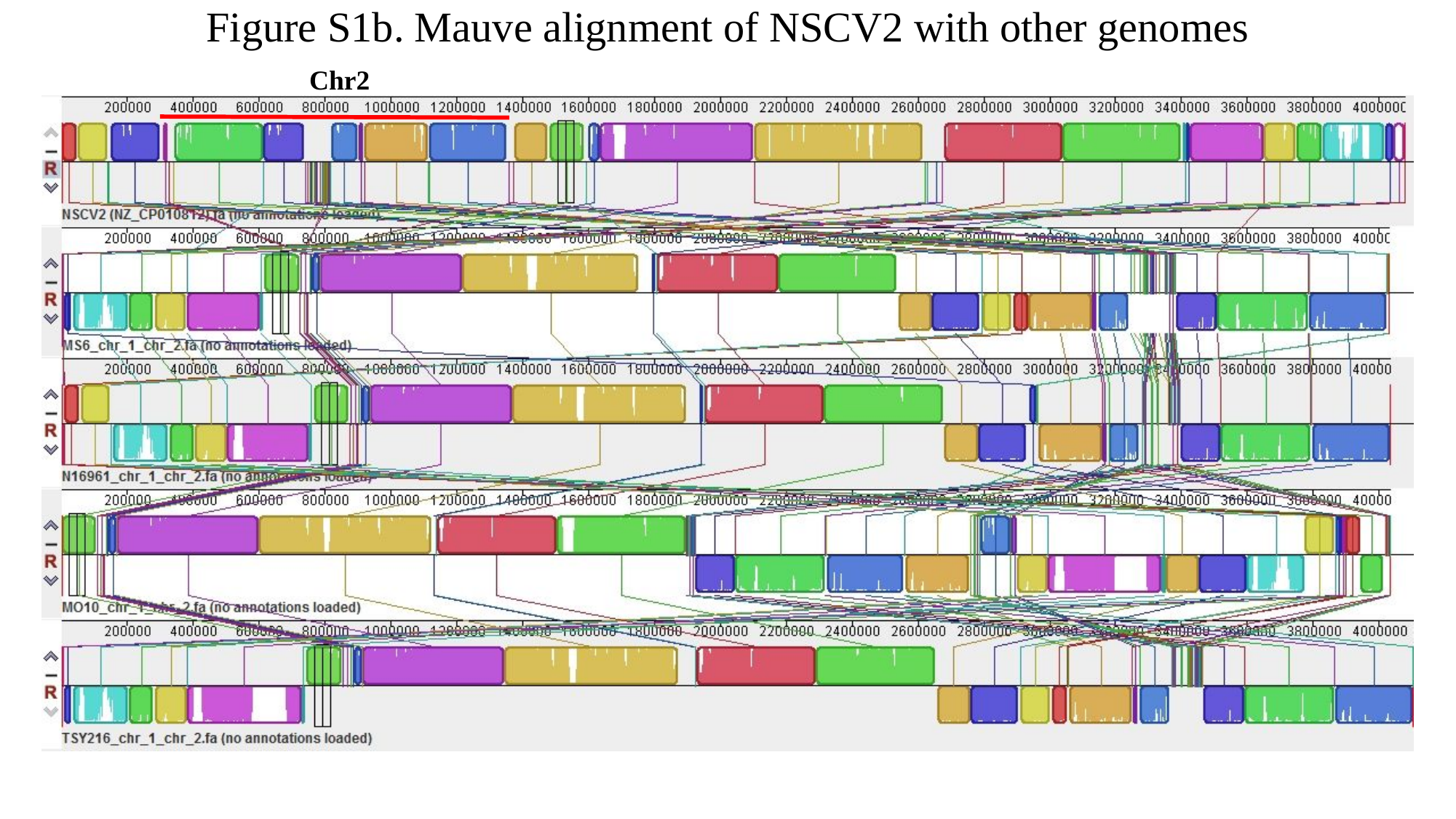

Figure S1b. Mauve alignment of NSCV2 with other genomes
Chr2

## Slide 3
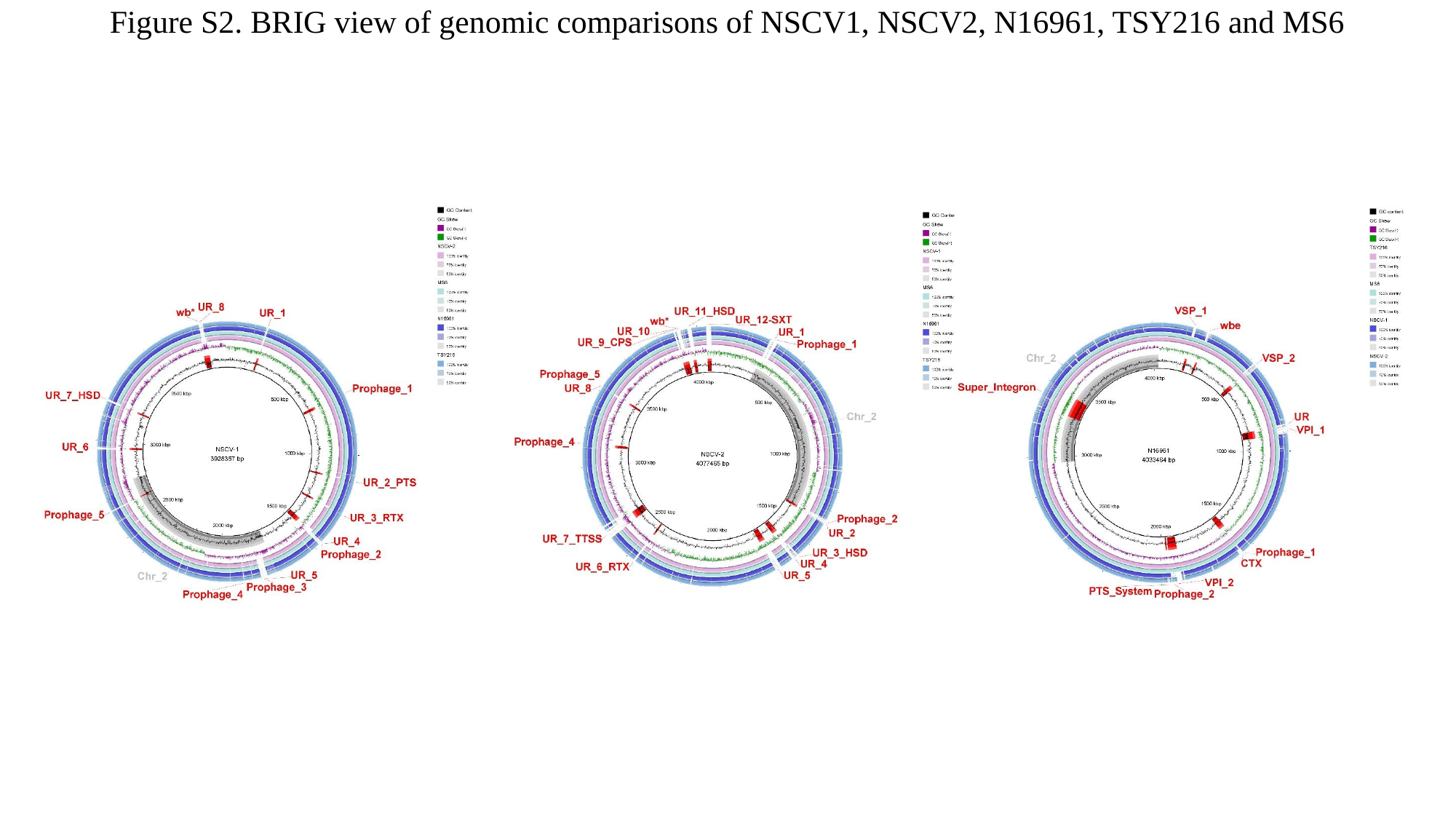

Figure S2. BRIG view of genomic comparisons of NSCV1, NSCV2, N16961, TSY216 and MS6

## Slide 4
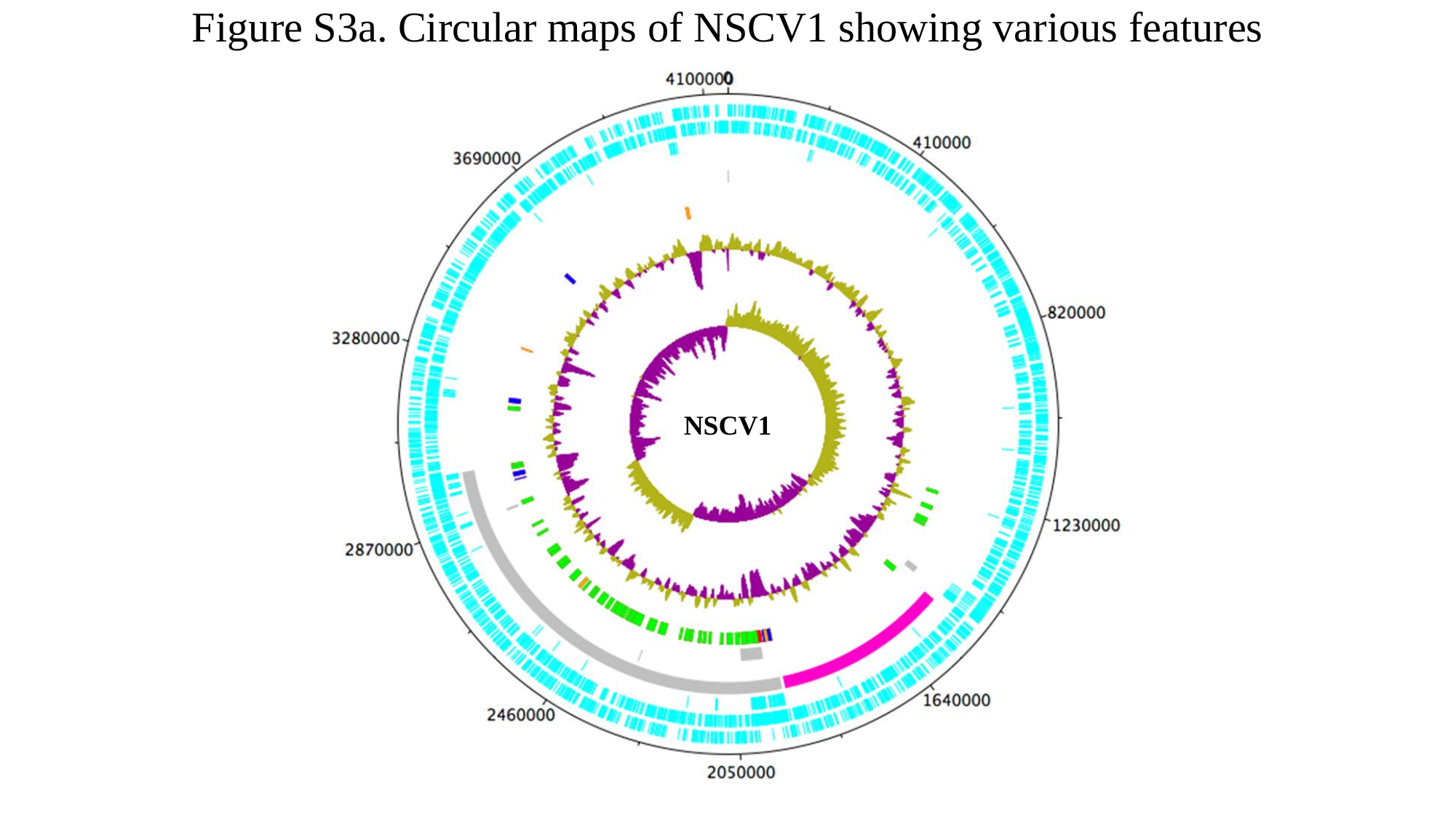

Figure S3a. Circular maps of NSCV1 showing various features
NSCV1

## Slide 5
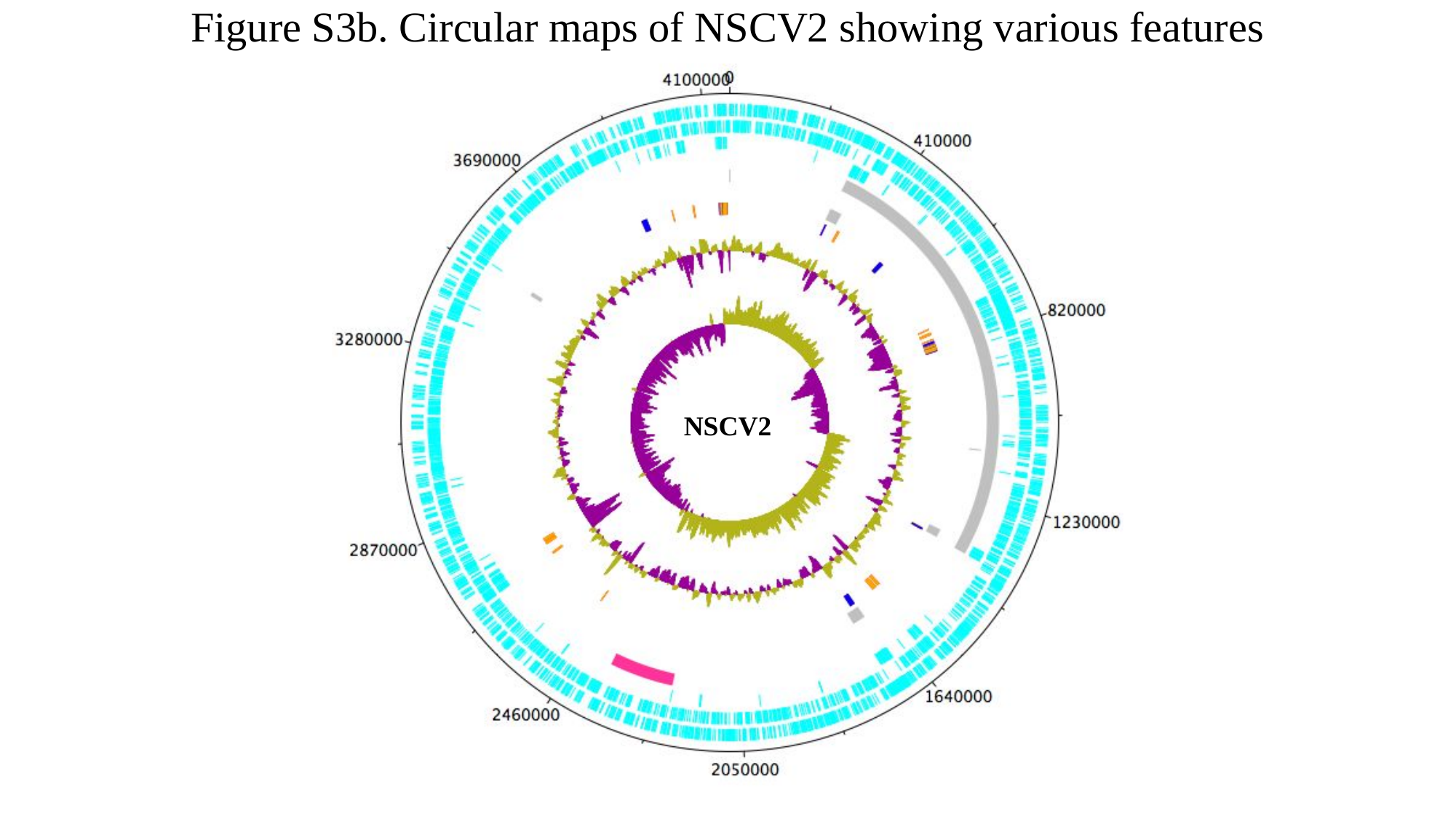

Figure S3b. Circular maps of NSCV2 showing various features
NSCV2

## Slide 6
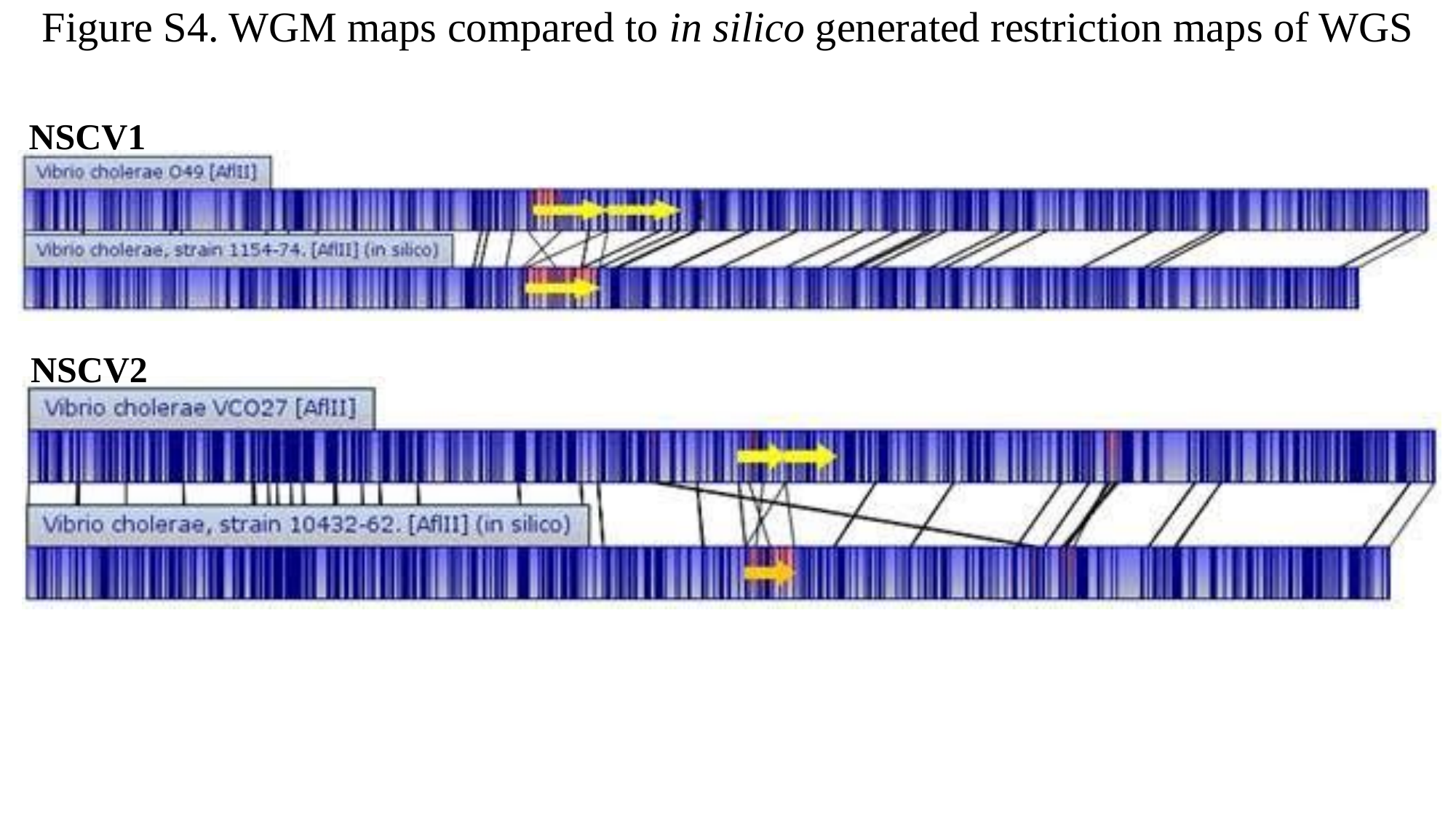

Figure S4. WGM maps compared to in silico generated restriction maps of WGS
NSCV1
NSCV2

## Slide 7
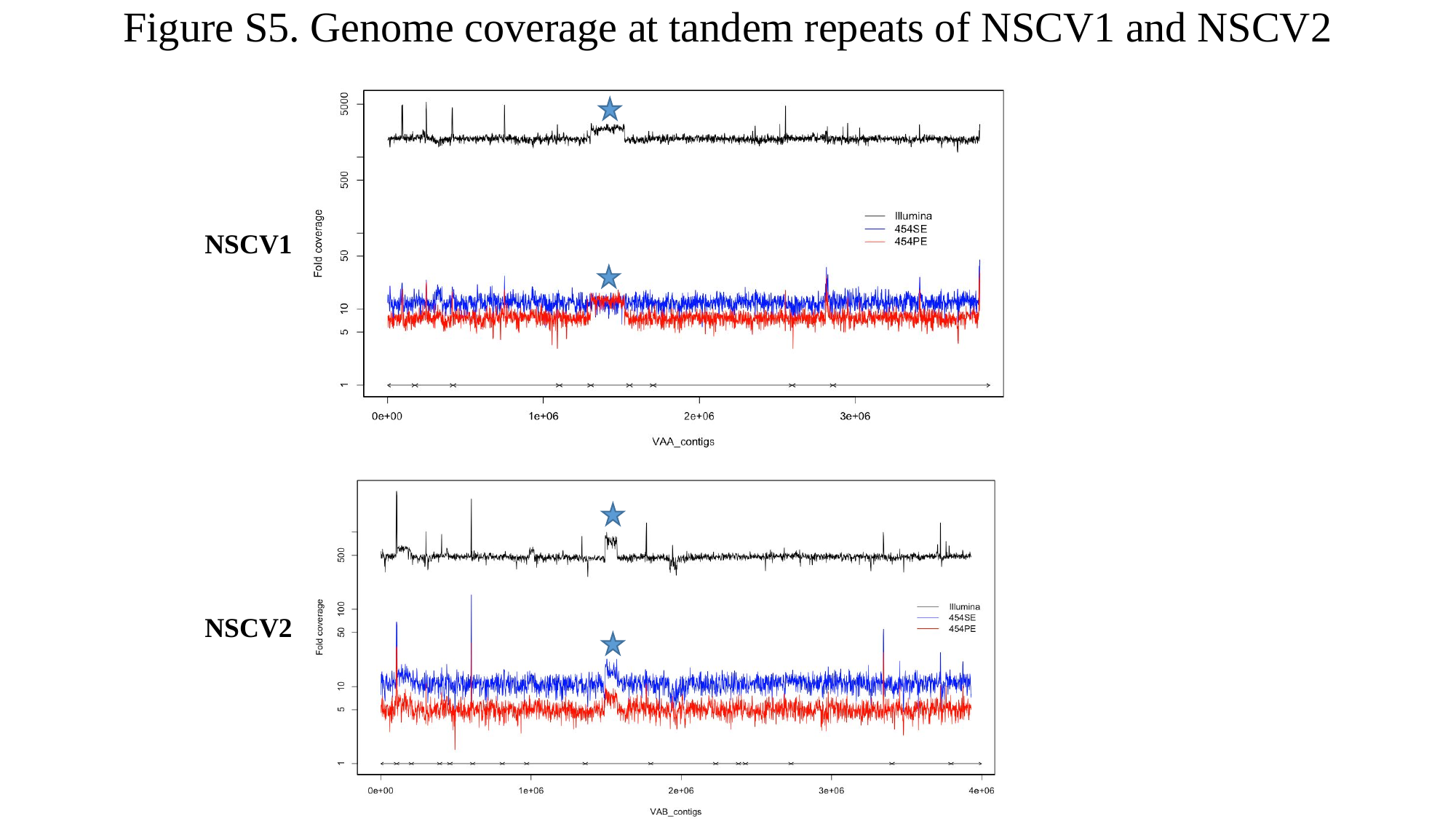

Figure S5. Genome coverage at tandem repeats of NSCV1 and NSCV2
NSCV1
NSCV2

## Slide 8
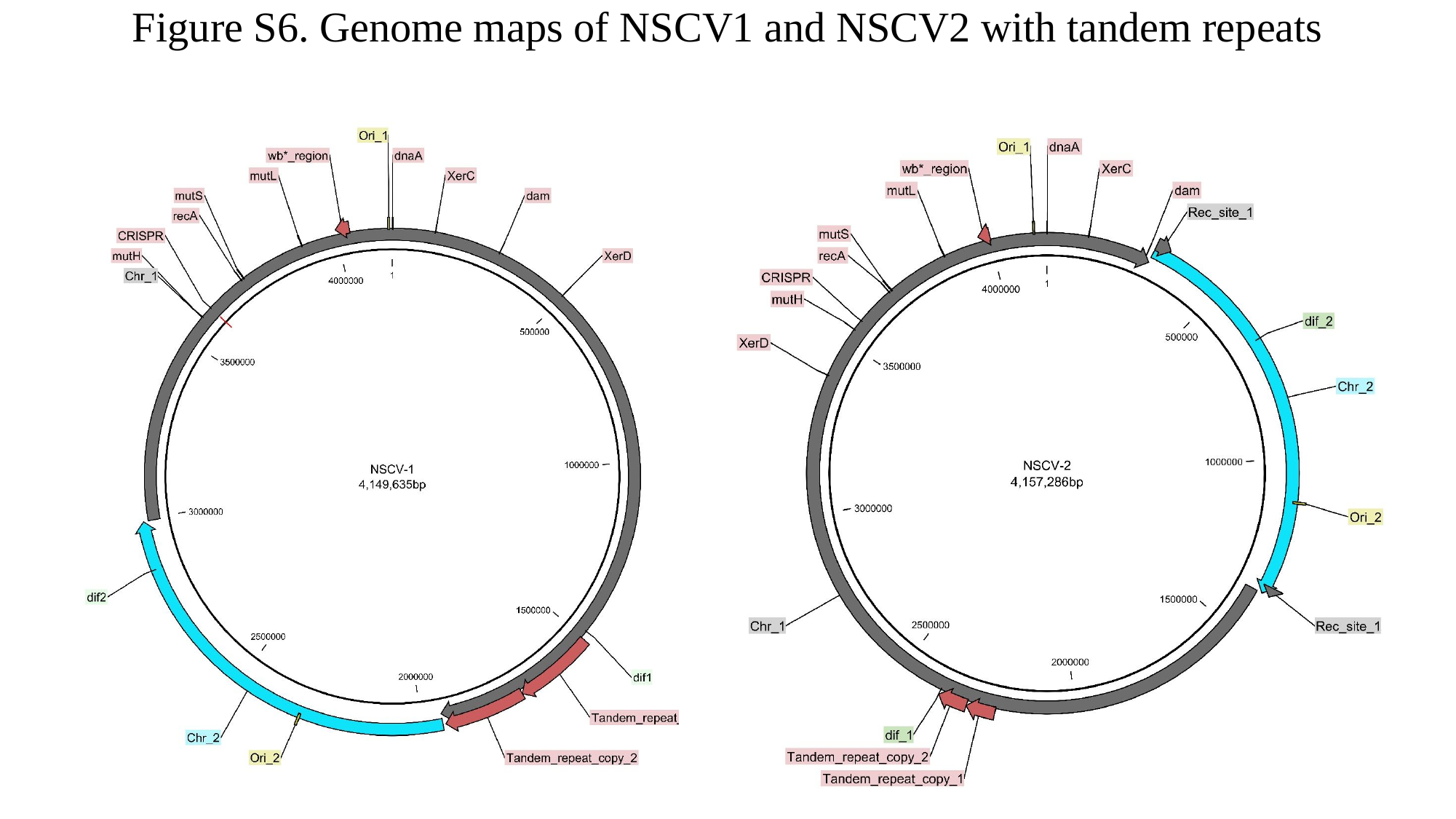

Figure S6. Genome maps of NSCV1 and NSCV2 with tandem repeats

## Slide 9
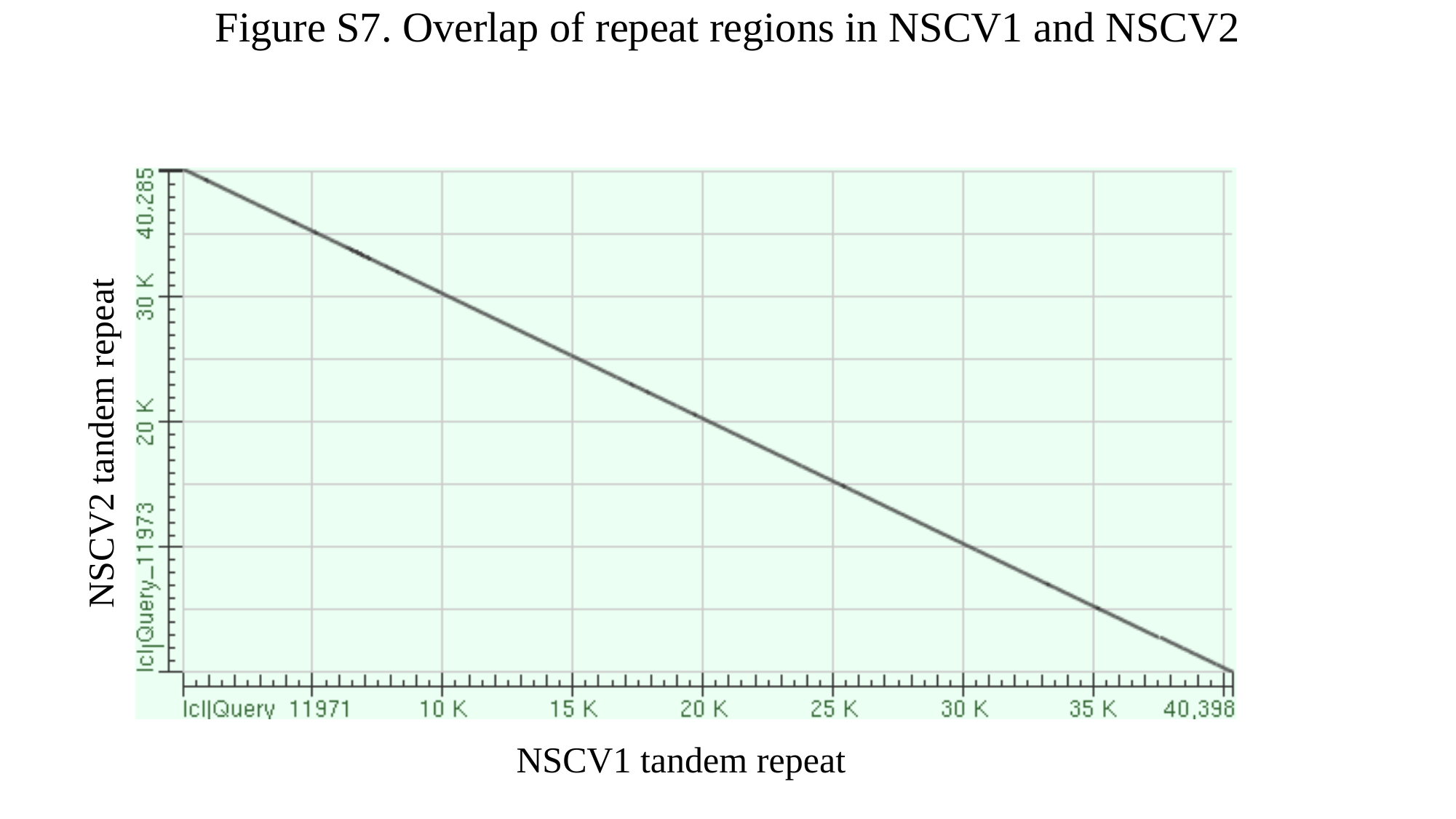

Figure S7. Overlap of repeat regions in NSCV1 and NSCV2
NSCV2 tandem repeat
NSCV1 tandem repeat

## Slide 10
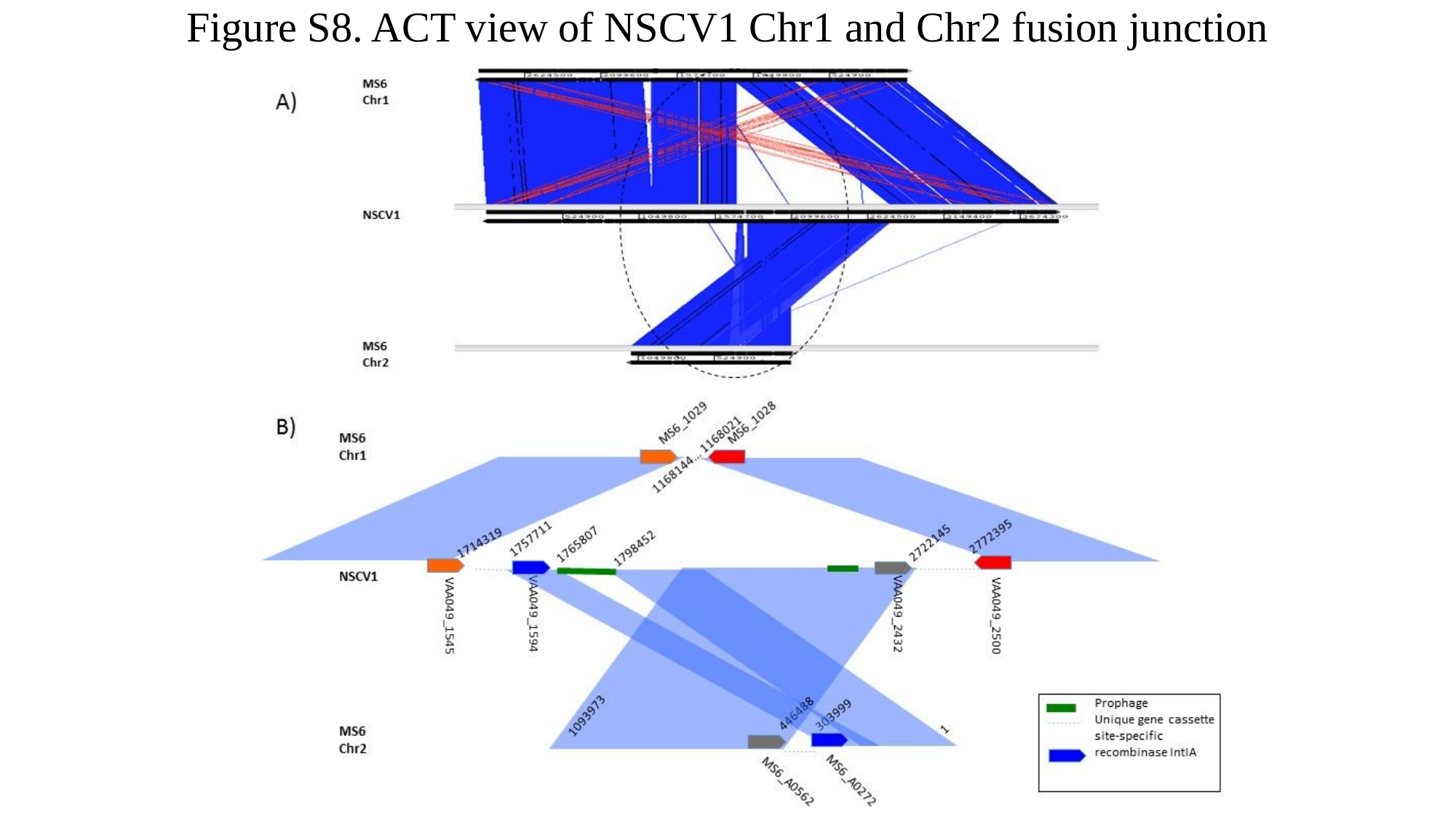

Figure S8. ACT view of NSCV1 Chr1 and Chr2 fusion junction

## Slide 11
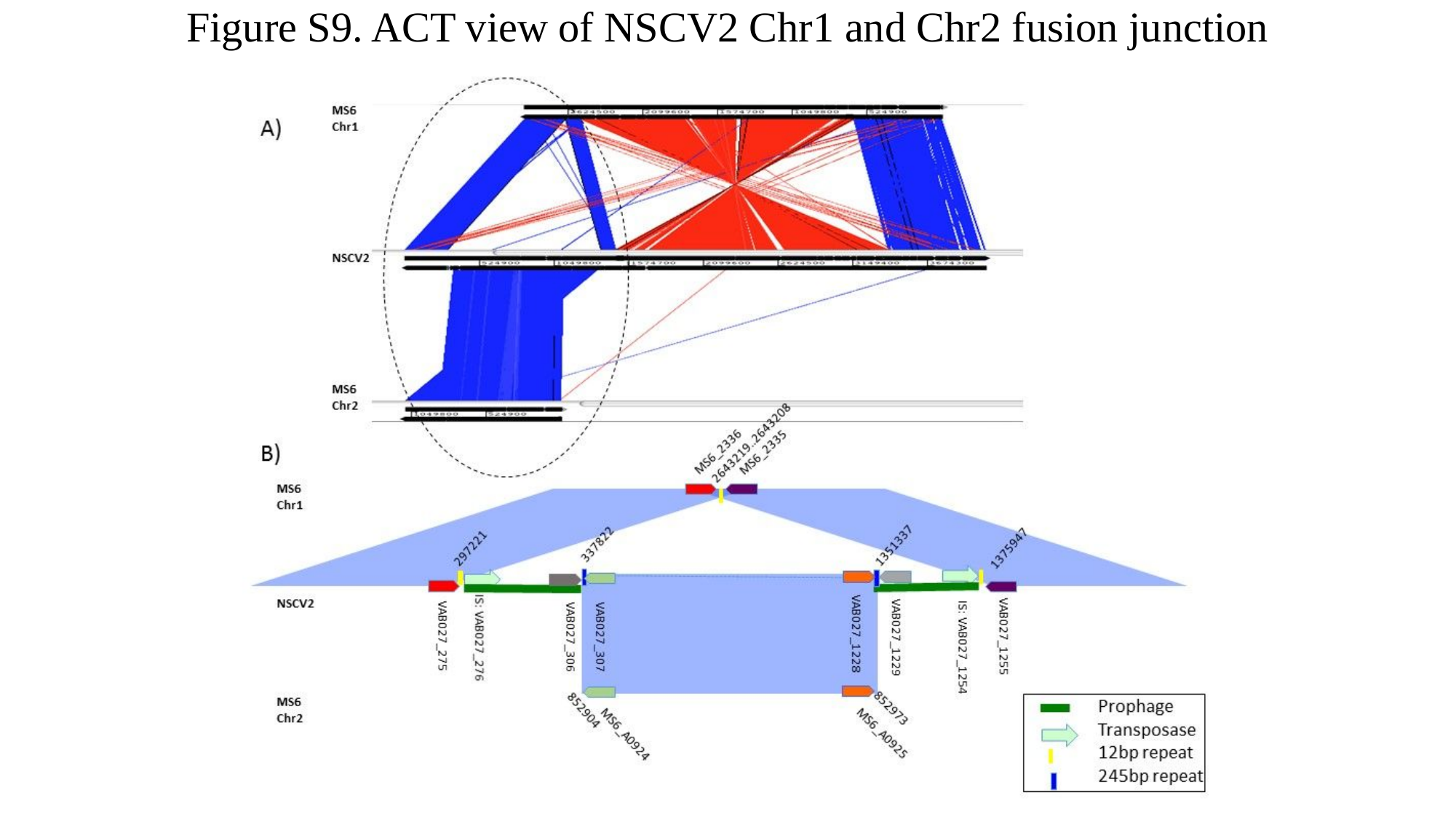

Figure S9. ACT view of NSCV2 Chr1 and Chr2 fusion junction
