# Supplementary material for: Exception to the Rule: Genomic Characterization of Naturally Occurring Unusual Vibrio cholerae Strains with a Single Chromosome
Source: Int J Genomics. 2017 Aug 29;2017:8724304. doi: 10.1155/2017/8724304 (PMC5603330; doi:10.1155/2017/8724304)
Supplement: Supplementary file 3 [file 8724304.f3.docx]

**Table S4. List of potential *dif* sites in NSCV1 and NSCV2**

**Dif-site alignment: Location**

E. coli GGTGCGCATAATGTATATTATGTTAAAT

N16961 chrII AATGCGCATTACGTGCGTTATGTTAAAT

N16961 chrI AGTGCGTATTATGTATGTTATGTTAAAT

Vibrio_1154-74 (VAA) AGTGCGTATTAGGTATATTATGTTAAAT (1,476,590)

Vibrio_1154-74 (VAA) AGTACATATTATGTATGTTATGTTAAAT (2,643,791)

Vibrio_10432-62 (VAB) ACTTCGTATTACGTGTGTTATGTTAAAT (664,646)

Vibrio_10432-62 (VAB) AGTGCGTATTAGGTATATTATGTTAAAT (2,301,152)

**ChrI-dif alignment:**

N16961 chrI AGTGCGTATTATGTATGTTATGTTAAAT

Vibrio_1154-74 (VAA) AGTGCGTATTAGGTATATTATGTTAAAT (1,476,590)

Vibrio_10432-62 (VAB) AGTGCGTATTAGGTATATTATGTTAAAT (2,301,152)

**ChrII-dif alignment:**

N16961 chrII AATGCGCATTACGTGCGTTATGTTAAAT

Vibrio_1154-74 (VAA) AGTACATATTATGTATGTTATGTTAAAT (2,643,791)

Vibrio_10432-62 (VAB) ACTTCGTATTACGTGTGTTATGTTAAAT (664,646)
