# Supplementary material for: Exception to the Rule: Genomic Characterization of Naturally Occurring Unusual Vibrio cholerae Strains with a Single Chromosome
Source: Int J Genomics. 2017 Aug 29;2017:8724304. doi: 10.1155/2017/8724304 (PMC5603330; doi:10.1155/2017/8724304)

Supplementary Tables

Table_S1. Whole Genome Sequence Statistics of NSCV1 and NSCV2								
**Strain**	**NSCV-1 ((1154-74 (O49) VAA))**				**NSCV-2 ((10432-62 (O27) VAB))**			
Sequencing platform	Roche 454 (a)	Roche 454 (b)	Illumina	PacBio RS	Roche 454 (a)	Roche 454 (b)	Illumina	PacBio RS
Sequencing detail	Titanium	Titanium	GA iiX	P4-C2	Titanium	Titanium	GA iiX	P5-C3
Library Type	Fragments	Long-Insert Pairs	Fragments	Fragments	Fragments	Long-Insert Pairs	Fragments	Fragments
Assembly Statistics								
Mean Read Length (bps)	306	164	100	5,966	396	167	76	4,458
Total Reads	307,619	286,661	82,893,269	145,955	219,494	145,870	26,677,286	340,387
Total bps	94,151,248	47,134,856	8,289,326,900	870,847,956	86,989,637	24,404,545	2,027,473,736	1,517,667,722
Average Insert size	n/a	9,528 ± 2,382	n/a	n/a	n/a	5,340 ± 1,335	n/a	n/a
Total PE reads	n/a	124,071	n/a	n/a	n/a	57,858	n/a	n/a
Depth of coverage (×)	21		2,178	201	17		507	350
Genomic features of the final sequence								
SRA Accession Number	SPR061796				SPR061797			
GenBank Accession Number	NZ_CP010811.1				NZ_CP010812.1			
Total Genome size (bps)	3,928,357				4,077,462			
GC content (%)	47.8				47.7			
Genes	3,641				3,795			
Coding DNA Sequences	3,458				3,585			
CDS with assigned function	2,882				2,894			
Percentage of CDS with assigned function	83.3				80.7			
# hypothetical proteins	576				691			
Pseudo Genes	41				89			
# rRNA	31				22			
# tRNA	104				94			
ncRNA	1				1			
Frameshifted Genes	27				56			
Frameshifted Genes On Monomer Runs	6				9			
Frameshifted Genes Not on Monomer Runs	2				0			
Optical Map Estimate	3,889,393				3,801,481			

Table_S2. Nucmer based pairwise comparison of genome sequences

Query\ref	NSCV1	NSCV2	N16961	TSY216	MS6	MO10
NSCV1		11.61/88.39	8.33/91.67	26.43/73.57	8.61/91.84	10.61/89.39
NSCV2	7.50/92.50		8.70/91.30	26.66/73.34	8.53/91.47	10.82/89.18
N16961	5.70/94.30	9.96/90.04		19.96/80.04	1.23/98.77	4.35/95.65
TSY216	5.81/94.19	10.01/89.99	0.37/99.63		1.44/98.56	2.93/97.07
MS6	5.63/94.37	10.04/89.96	1.40/98.60	20.92/79.08		5.52/94.48
MO10	6.34/93.66	10.40/89.60	2.20/97.80	20.15/79.85	3.18/96.82	

Two way pairwise comparison and percentage of unique/shared sequences between the genome sequences

Table_S3. Unique Regions ~10 Kb and over in NSCV1, NSCV2 and N16961 in comparison to others genomes														
**NSCV1**					**NSCV2**					**N16961-Chr_1 and**				
Feature	Annotatio	Start	End	Size	Feature	Annotatio	Start	End	Size	Feature	Annotatio	Start	End	Size
1	UR_1	193223	203529	10306	1	UR_1	297222	337551	40329	1	VSP-1	175198	189459	14261
2	Prophage_	685729	702622	16893	2	Prophage_	299327	349041	49714	2	wbe	249000	260427	11427
3	UR_2_PT	1121690	1131437	9747	3	Chr_2	297221	1375947	1078726	3	VSP-2	522877	550186	27309
4	UR_3_RT	1290731	1301721	10990	4	Prophage_	1359430	1369981	10551	4		865209	874265	9056
5	UR_4	1446108	1476589	30481	5	UR_2	1359610	1370754	11144		VPI-1	875011	914239	39228
6	Prophage_	1458739	1476484	17745	6	UR_3_HS	1575177	1585466	10289	5	VPI-2	1895816	1953315	57499
7	UR_5	1765808	1798451	32643	7	UR_4	1587780	1610634	22854	6	PTS Syste	1960947	1970701	9754
8	Prophage_	1756187	1818403	62216	8	UR_5	1676773	1710753	33980	7	Chr_1	1-2961149		2961149
9	Prophage_	1879405	1883642	4237	9	Prophage_	1676903	1714350	37447	8	Ori_1	2961047-2	1-371	474
10	Chr_2	1720246	2772395	1052149	10	UR_6_RT	2451538	2462245	10707		Rep_Ori_	2956820-2	1-806	5136
11	Prophage_	2643793	2650802	7009	11	UR_7_TT	2616566	2665918	49352	9	Chr_2	2961150-4033464		1072315
12	UR_6	2955772	2968930	13158	12	Prophage_	3113341	3130219	16878	10	Ori_2	2961396-2962282		887
13	UR_7_HS	3195919	3205927	10008	13	UR_8	3416243	3427115	10872		Rep_Ori_	2961150-2	4030845-4	5811
14	wb*_regio	3777887	3815081	37194	14	Prophage_	3416918	3428588	11670	11	Prophage_	1546757	1577272	30515
15	UR_8	3783110	3812259	29149	15	UR_9_CP	3878812	3888045	9233	12	Prophage_	1935801	1946144	10343
					16	UR_10	3892943	3914220	21277	13	Super Inte	3270899	3396567	125668
					17	wb*_regio	3888709	3920844	32135					
					18	UR_11_H	3949310	3969802	20492					
					19	UR_12_S	4044299	4070910	26611					

Table S4. List of potential *dif* sites in NSCV1 and NSCV2

```
Dif-site alignment:                                           Location
E. coli                 GGTGCGCATAATGTATATTATGTTAAAT
N16961 chrII            AATGCGCATTACGTGCGTTATGTTAAAT
N16961 chrI             AGTGCGTATTATGTATGTTATGTTAAAT
Vibrio_1154-74 (VAA)   AGTGCGTATTAGGTATATTATGTTAAAT (1,476,590)
Vibrio_1154-74 (VAA)    AGTACATATTATGTATGTTATGTTAAAT (2,643,791)
Vibrio_10432-62 (VAB)  ACTTCGTATTACGTGTGTTATGTTAAAT (664,646)
Vibrio_10432-62 (VAB)   AGTGCGTATTAGGTATATTATGTTAAAT (2,301,152)


ChrI-dif alignment:
N16961 chrI             AGTGCGTATTATGTATGTTATGTTAAAT
Vibrio_1154-74 (VAA)   AGTGCGTATTAGGTATATTATGTTAAAT (1,476,590)
Vibrio_10432-62 (VAB)  AGTGCGTATTAGGTATATTATGTTAAAT (2,301,152)


ChrII-dif alignment:
N16961 chrII            AATGCGCATTACGTGCGTTATGTTAAAT
Vibrio_1154-74 (VAA)    AGTACATATTATGTATGTTATGTTAAAT (2,643,791)
Vibrio_10432-62 (VAB)  ACTTCGTATTACGTGTGTTATGTTAAAT (664,646)
```

Table_S5. Summary of nucleotide and amino acid changes in origins of replication genes and corresponding proteins													
Summary of nucleotide changes								Summary of amino acid changes					
				Same alleles							Same alleles		
Locus ID in O1	Gene	Size	No of mutations	O1/O49	O1/O27	O27/O49	Different alleles#	Protein	Size	Position	N16961_O1	1154-74_O49	10432-62_O27
	OriI	5136*		42	41	33		ParB	293	35	A	V	A
VC2772	*parB*	882	8	2	4	2				103	Q	L	Q
VC2773	*parA*	774	6	0	1	5		ParA	257	1	V	M	M
VC2774	*gidB*	633	12	2	4	6				25	A	S	A
VC2775	*gidA*	1896	21	9	3	9		GidB	210	77	S	T	T
	oriCI	474	8	1	4	3				98	I	L	L
VC0002	*mioC*	435	3	2	1	0				162	A	A	G
	Total		58	16	17	25		GidA	631	361	Y	F	F
										464	A	A	V
								MioC	144	None			
	OriII	5811**		65	58	59							
VCA1113	*VCA1113*	263	5	0	2	3		VCA1113	87	None			
	ig	59	3	0	3	0		ParB	324	None			
VCA1114	*parB*	972	9	1	6	2		ParA	406	None			
VCA1115	*parA*	1218	9	2	5	2		RctA	45	19	R	Q	R
	ig1	143	6	3	3	0				31	I	N	I
VCA0001	*rctA*	135	2	0	2	0		RctB	659	393	T	A	A
	ig2	887	15	4	2	8	1			433	D	E	D
VCA0002	*rctB*	1977	41	15	9	16	1						
	Total		90	25	32	31							
* Δ1 bp in OriCI in O49													
** 1 bp insertion in O49, 2 bps deletion in [illegible]													
#Different alleles in each strain													

Table_S6. Summary of nucleotide and amino acid changes in replication associated and mismatch repa

Summary of nucleotide changes								Summary of amino acid changes					
				Same alleles							Same alleles		
Locus ID in O1	Gene	Size	No of mutations	O1/O49	O1/O27	O27/O49	Different alleles[#]	Protein	Size	Position	N16961_O1	1154-74_O49	10432-62_O27
	dnaA	1419	64	0	Δ 1-15			DnaA	472	3-7	WT	ΔEGIVS	
				28	5	16							
VC2626	*dam*	834	12	5	2	5		Dam	277	0			
VC0543	*recA*	1065	34	9	8	17		RecA	354	305	Y	C	C
VC0668	*mutH*	666	12	2	6	4		MutH	221	158	W	L	W
VC0345	*mutL*	1962	25	12	3	10				200	A	T	T
VC0535	*mutS (O1)*	2589	94	24	36	33	1	MutL	653	46	L	I	I
	mutS (O27)	2579	Δ (386-395)							90	M	V	M
VC0128	*xerC*	936	13	2	4	7				281	V	A	A
VC2419	*xerD*	909	19	4	7	8				332	G	S	S
										363	D	E	E
										396	P	Q	Q
										448	P	A	P
										465	I	I	T
								MutS (O1/O49)	862	0			
								MutS (O27)	842	11	S	S	Y
										129	L	L	R
										130	L	L	E
										132-150	WT	WT	Δ
										151	A	A	I
										236	K	K	Q
										408	D	N	D
										543	M	L	L
										582	E	K	E
								XerC	311	6	R	L	L
										103	Q	E	Q
										166	I	V	V
								XerD	302	0			

Supplemental Figure Legends

Figure S1. Mauve alignment of NSCV1 (S1a) and NSCV2 (S1b) with other genomes

NSCV1 and NSCV2 whole genome sequences were aligned to the genome sequences of *V. cholerae* strains MS6, N16961, MO10 and TSY216. The various collinear regions are indicated by different colored blocks. The Chr2 sections of NSCV1 and NSCV2 are indicated by a red line below the scale (bps). The large chromosomal inversion in NSCV2 (Figure S1b) can be seen as the blocks that are indicated on the opposite strand in MS6.

Figure S2. BRIG view of genomic comparisons of NSCV1, NSCV2, N16961, TSY216 and MS6. For genome comparisons, default blastn parameters were used. Unique regions (Uni_region) ~ 10 kb or more (along with annotation of the region if known) including prophages are indicated around the circle. In Figure 2c, using N16961 as the reference genome, Chr1 and Chr2 sequences are concatenated end to end and the various known virulence markers are indicated. N16961 is a prototypical *V. cholerae* with two chromosomes and the sequences are concatenated here for illustrative purpose only.

Figure S3. Circular maps of NSCV1 (S3a) and NSCV2 (S3b) showing various features

Circular map of the NSCV1 (S3a) and NSCV2 (S3b) genomes, showing the distribution of coding sequences, mobile elements, GC content and GC skew. For this analyses the sequences with large tandem repeats were included. From outside to the center: Circles 1 and 2: forward and reverse strand genes; Circle 3: unique genes in NSCV1 or NSCV2 in comparison with *V. cholerae* MS6, serogroup O1 biovar El Tor str. N16961, and serogroup O139 MO10; Circle 4: Chr2 in grey color and large tandem repeat in pink; Circle 5: OriC for Chr1 and 2; Circle 6: Prophage predicted by Phast; Circle 7: Genomic island Predicted by multiple methods: IslandPick, SIGI-HMM and IslandPath-DIMOB; Circle 8: GC content; Circle 9: GC skew.

Figure S4. WGM maps compared to *in silico* generated restriction maps of WGS

Whole genome optical maps (*Afl*II) of NSCV1 (top panel) NSCV 2 (bottom panel) aligned to *in silico* restriction maps (*Afl*II) using genome assemblies. The region of the sequence that appear as tandem repeats in the optical map are highlighted by the yellow arrows.

Figure S5. Genome coverage at tandem repeats of NSCV1 and NSCV2

Sequence reads of NSCV1 and NSCV 2 generated from Illumina and 454 sequencers were mapped against the respective final assemblies and displayed as function of fold coverage vs genome position in the assembly. Note that increased coverage indicated by the star is readily apparent except in the case of 454 SE read data for NSCV1. This anomaly could not be resolved at this time.

Figure S6. Genome maps of NSCV1 and NSCV2 with the large tandem repeats

Circular genome maps of NSCV1 and NSCV2 similar to Figure 1 with the tandem repeats included (red features). Fusion of Chr1 (dark grey) to Chr2 (blue) is shown in the circle at the respective locations. Various unique features such as tandem repeats, prophages and the origins of replication and replication associated genes are indicated around the circles.

Figure S7. Overlap of repeat regions between NSCV1 and NSCV2

The identity plot indicates the first 40,398 bps of NSCV1 tandem repeat region (total length of the repeat: 196,770 bps) which is 97% identical to the terminal 40,285 bps of NSCV2 tandem repeat region (total length of the repeat: 79,821 bps).

Figure S8. Artemis Comparison Tool (ACT) view of NSCV1 Chr1 and Chr2 fusion junction

ACT was used to compare the MS6 Chr1 and Chr2 sequences against NSCV1 sequence. Genome sequences were aligned from the predicted *ori1* and visualized in ACT with a cut-off set to blast scores >500. Red and blue bars indicate regions of similarity in the same orientation

(blue) and inverted (red). B) Zoom-in region of MS6-like Chr2 inserted in ChrI. All homologous genes at the insertion boundaries are the same color coded and the 2 prophages are indicated by the green bar.

Figure S9. Artemis Comparison Tool (ACT) view of NSCV2 Chr1 and Chr2 fusion junction

ACT was used to compare the MS6 Chr1 and Chr2 sequences against NSCV2. Genome sequences were aligned from the predicted OriC and visualized in ACT with a cut-off set to blast scores >500. Red and blue bars indicate regions of similarity in the same orientation (blue) and inverted (red) respectively. B) Zoom-in region of MS6 like Chr2 inserted in ChrI. All homologous genes at insertion boundaries are the same color coded. The green bars indicate the two prophages. The 12 bps and 245 bps repeats are labeled in yellow and blue line respectively.

Figure S1a. Mauve alignment of NSCV1 with other genomes

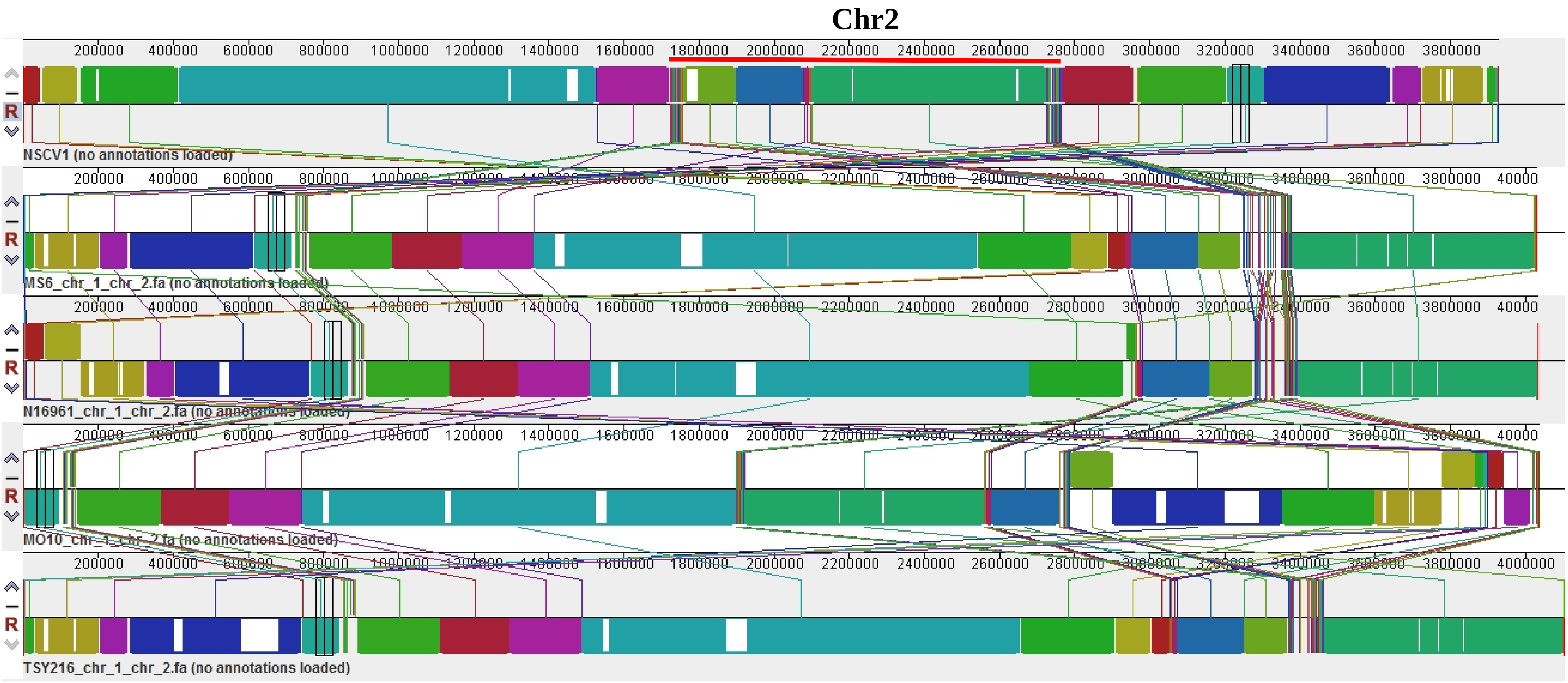

Figure S1b. Mauve alignment of NSCV2 with other genomes

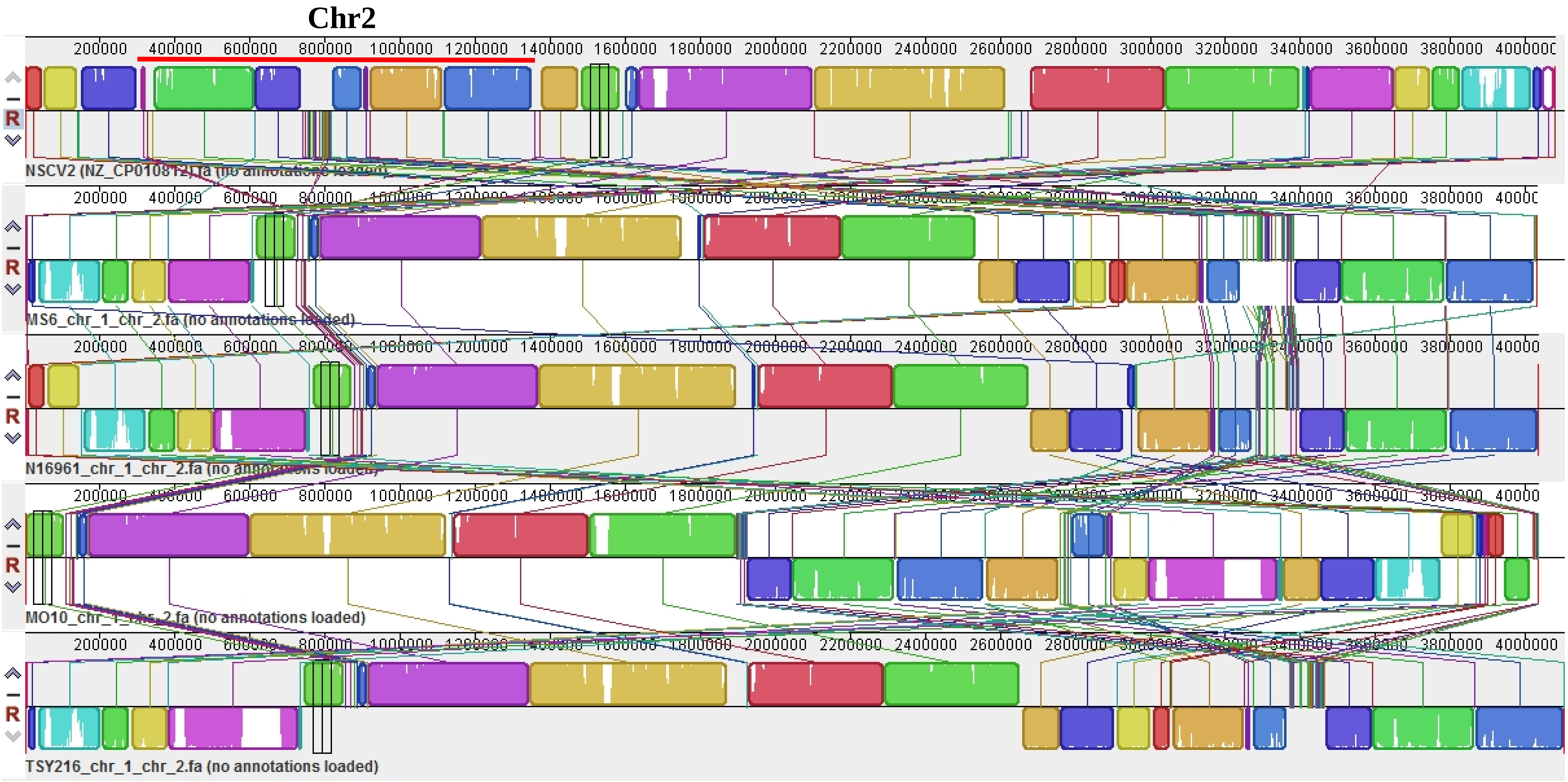

Figure S2. BRIG view of genomic comparisons of NSCV1, NSCV2, N16961, TSY216 and MS6

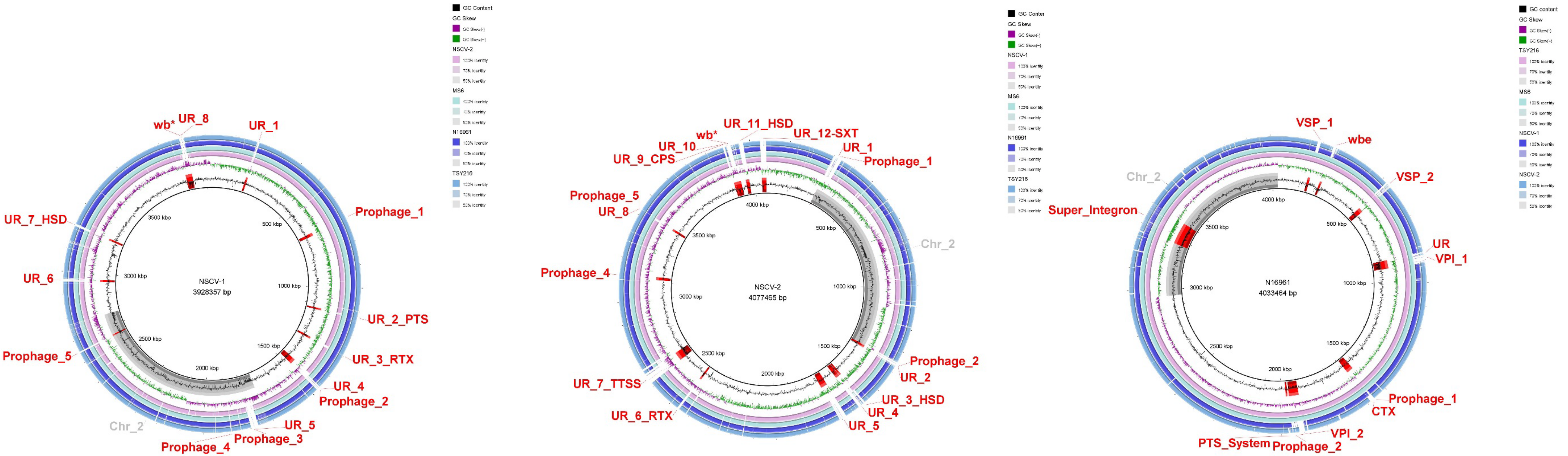

Figure S3a. Circular maps of NSCV1 showing various features

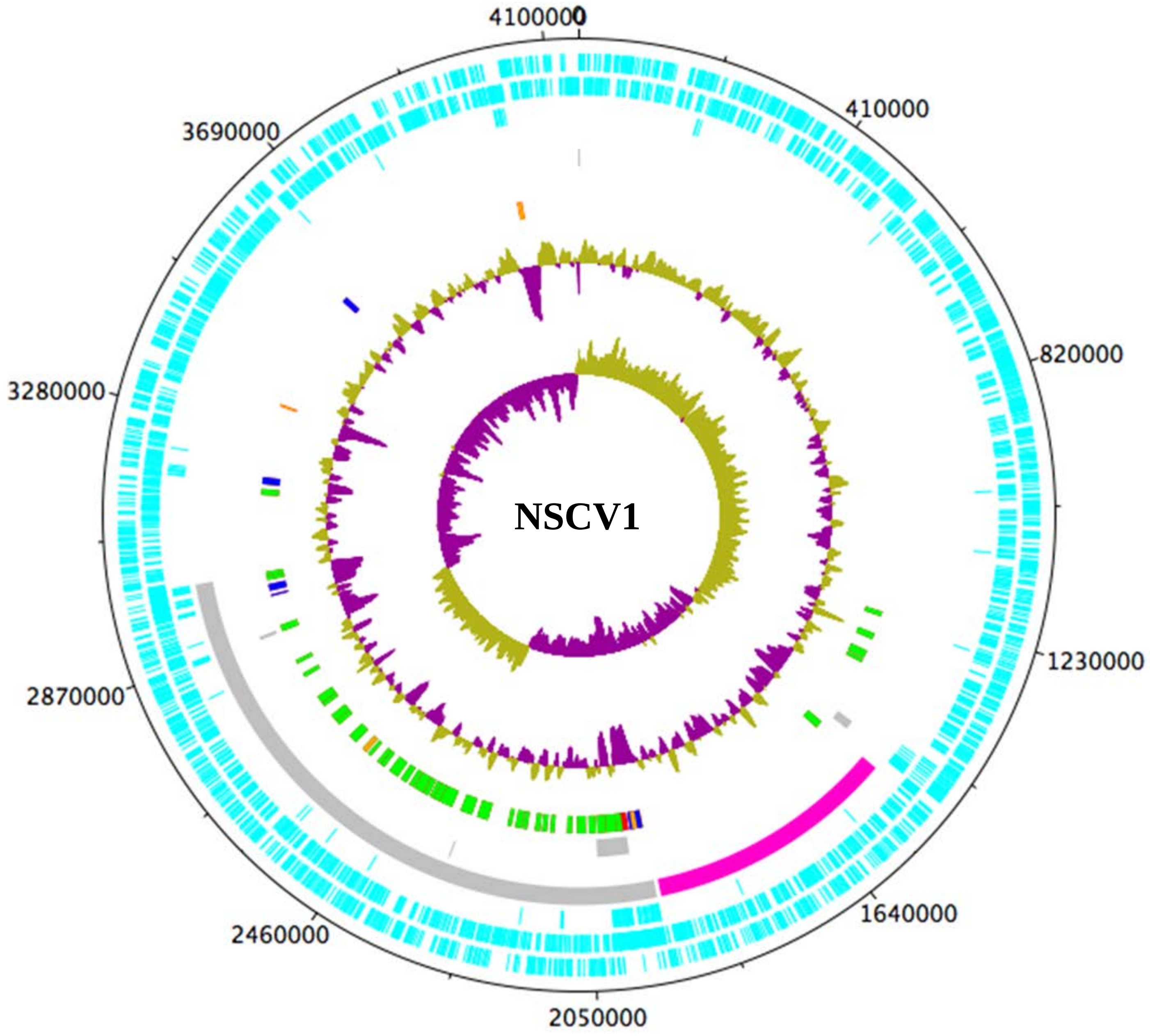

Figure S3b. Circular maps of NSCV2 showing various features

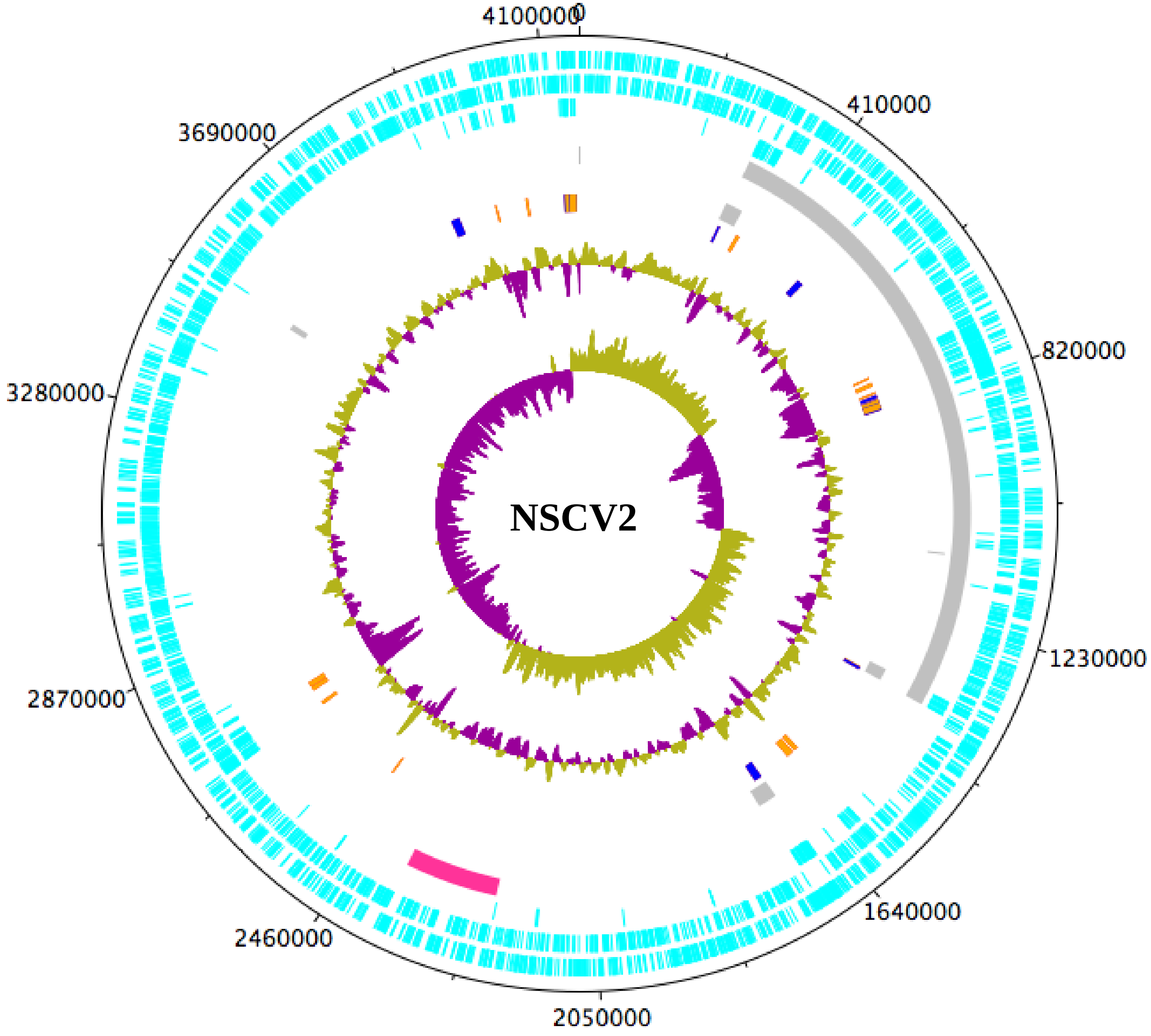

Figure S4. WGM maps compared to *in silico* generated restriction maps of WGS

NSCV1

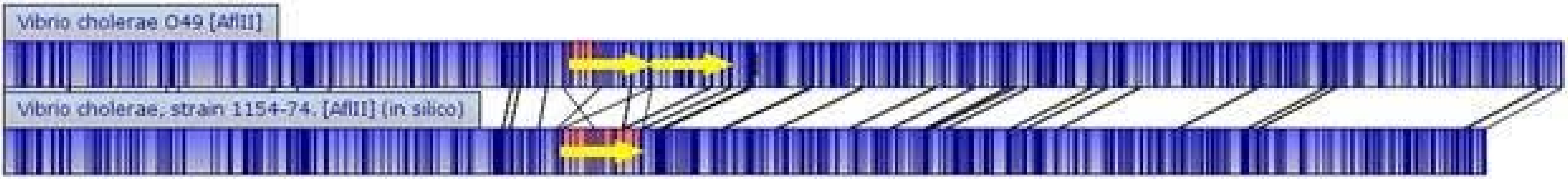

NSCV2

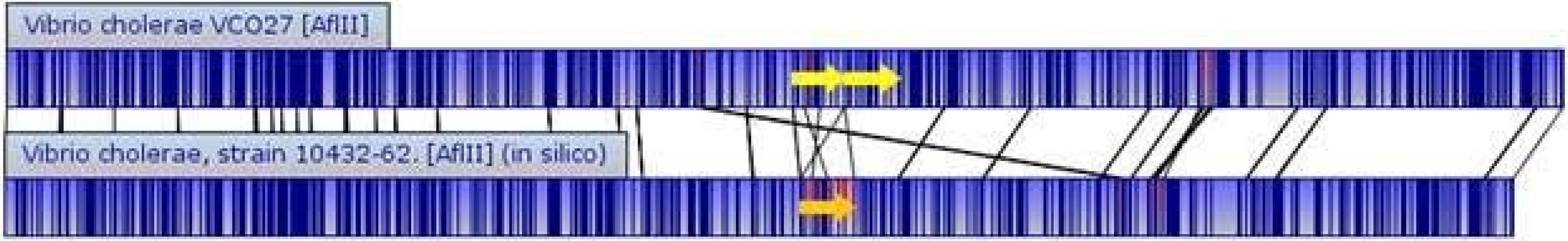

Figure S5. Genome coverage at tandem repeats of NSCV1 and NSCV2

NSCV1

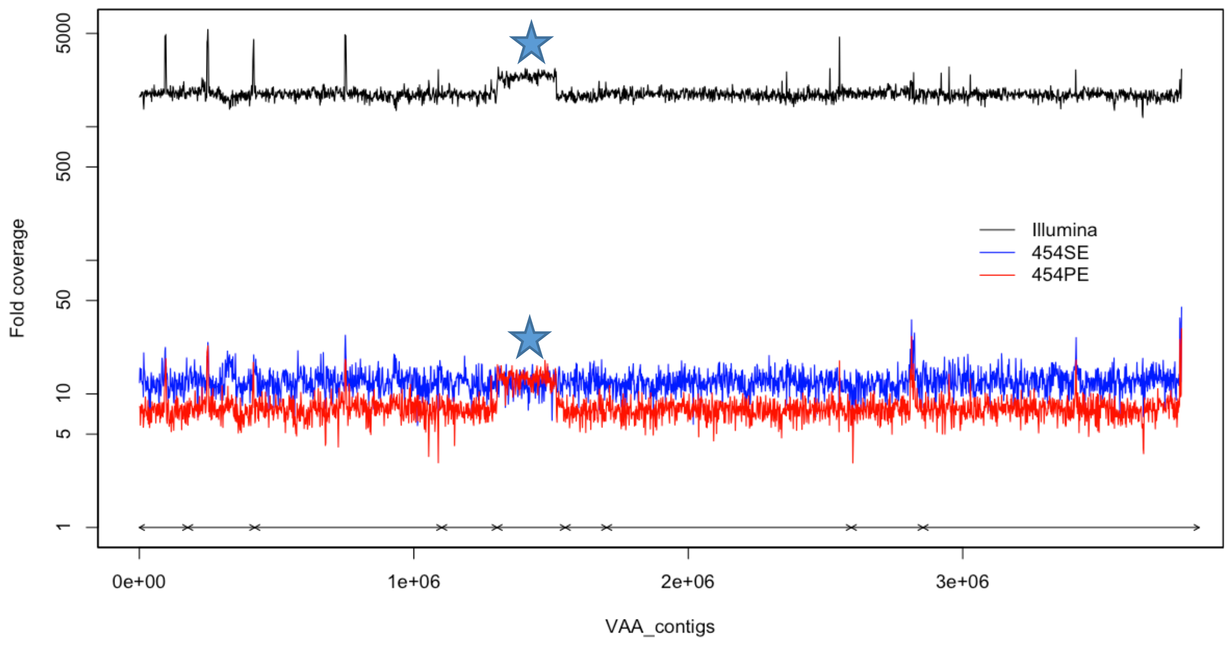

NSCV2

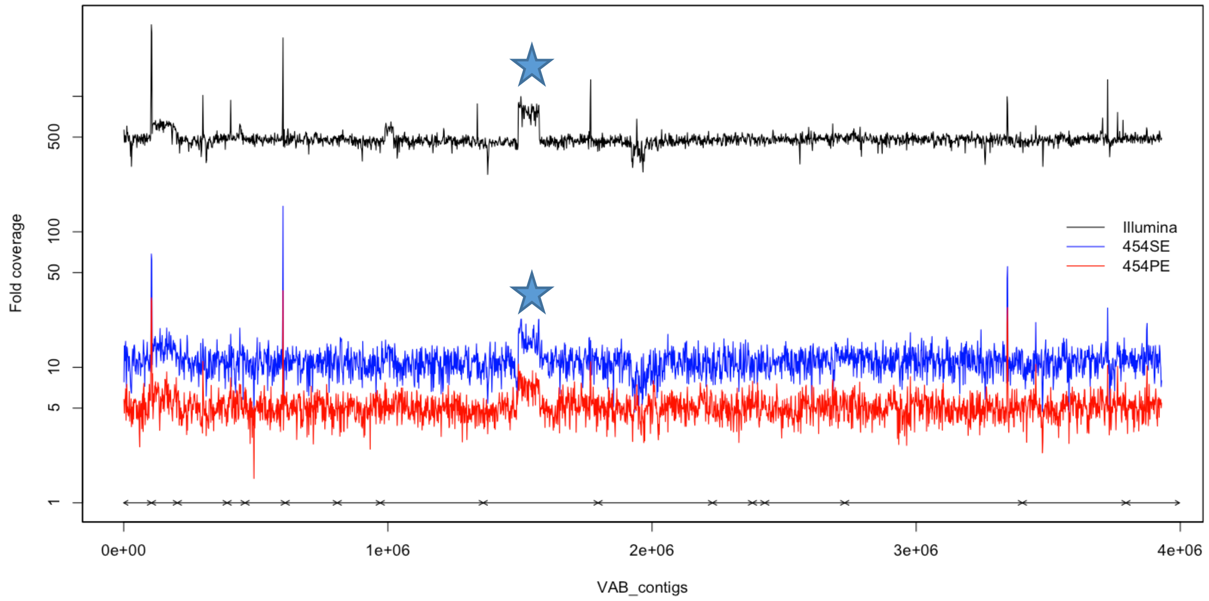

Figure S6. Genome maps of NSCV1 and NSCV2 with tandem repeats

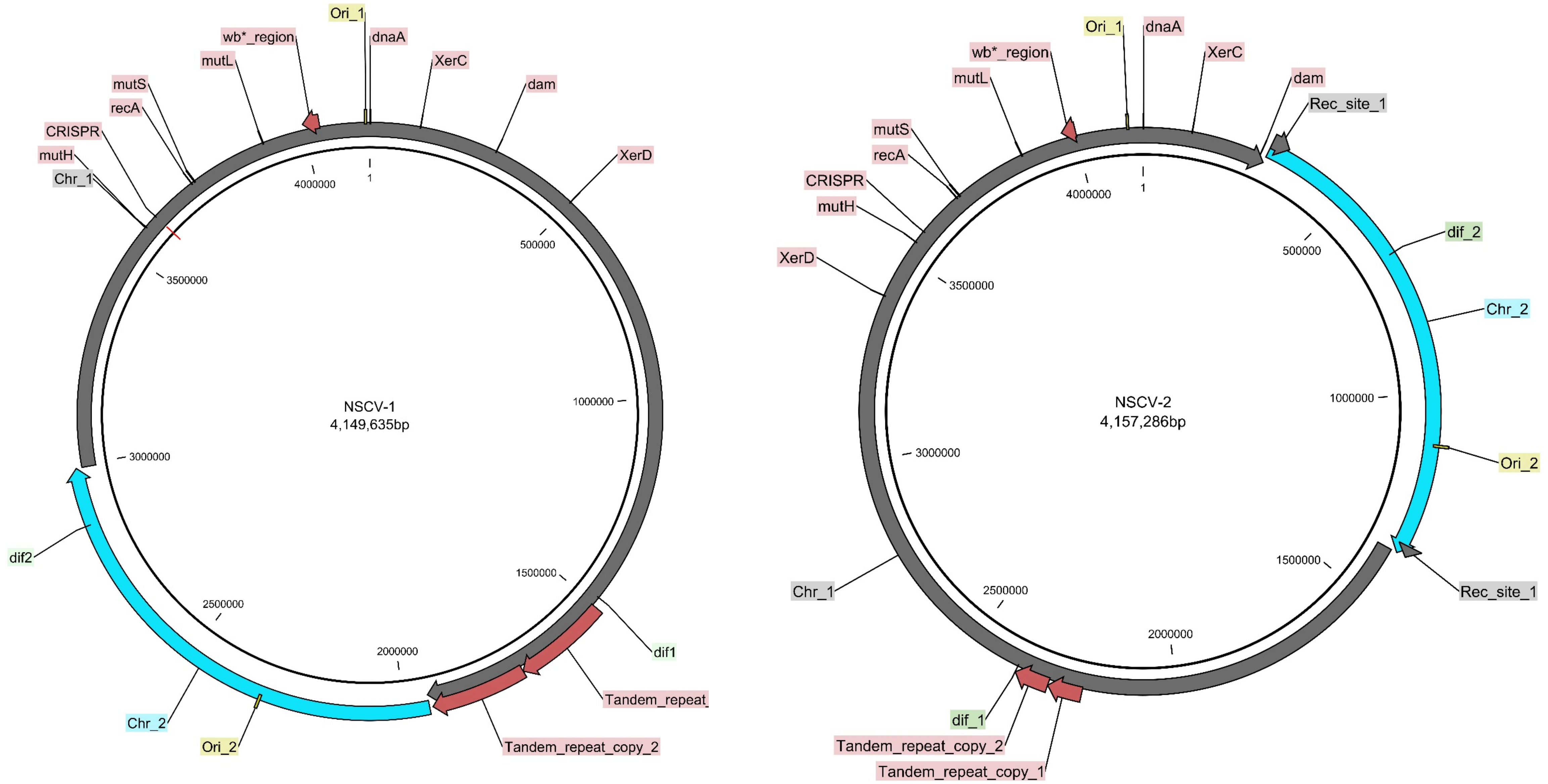

Figure S7. Overlap of repeat regions in NSCV1 and NSCV2

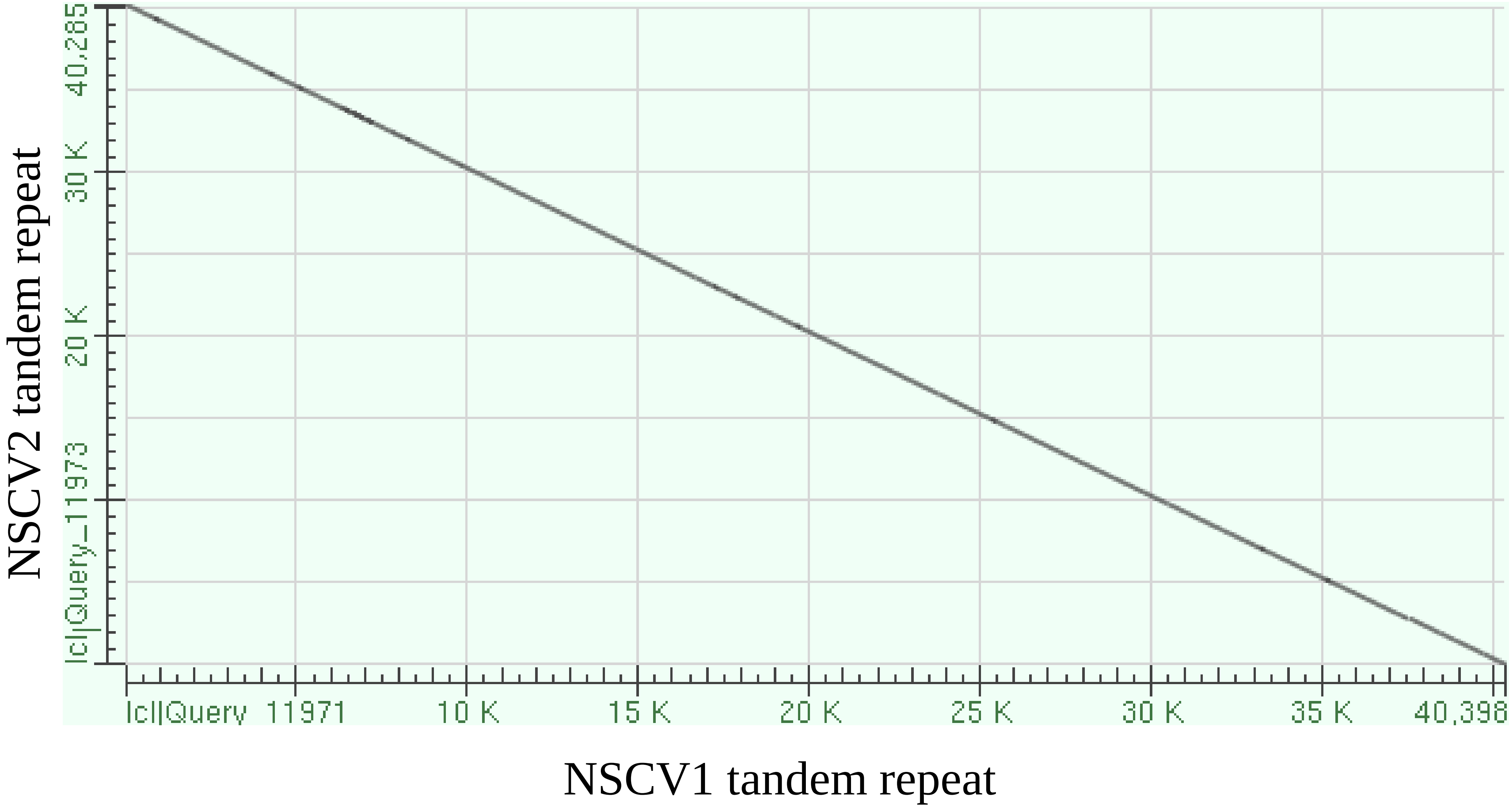

Figure S8. ACT view of NSCV1 Chr1 and Chr2 fusion junction

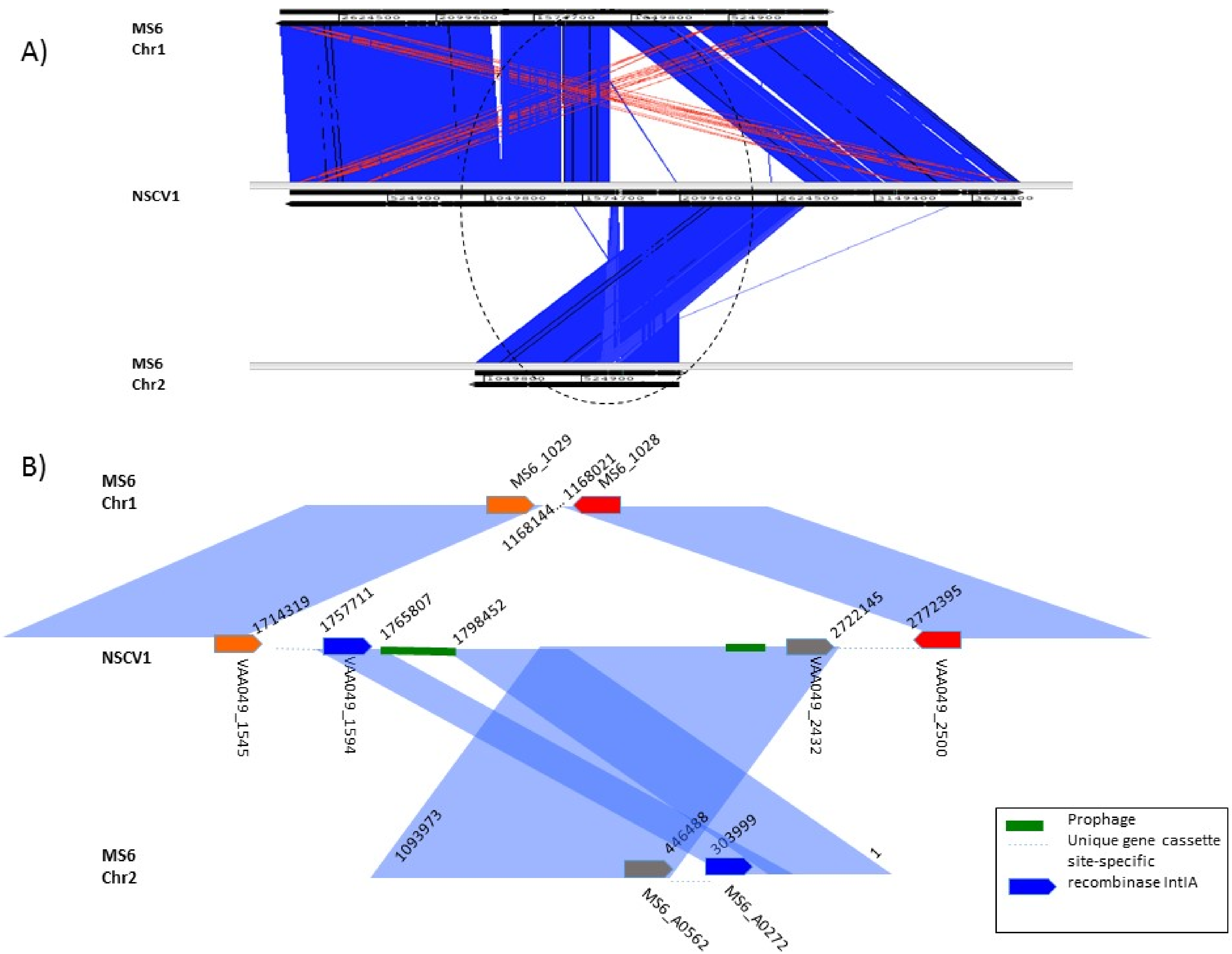

Figure S9. ACT view of NSCV2 Chr1 and Chr2 fusion junction

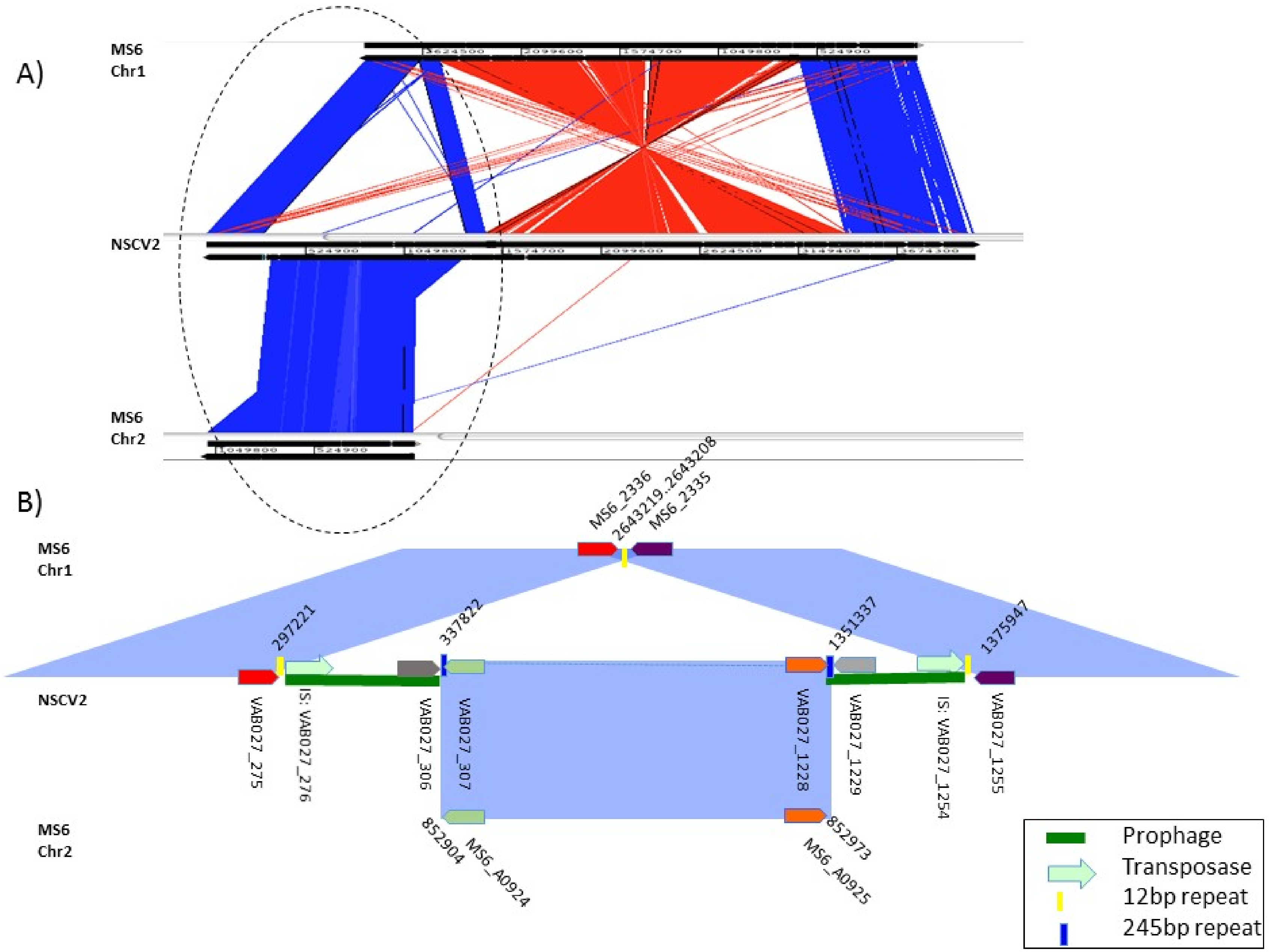

Supplement: Supplementary file 4 [file 8724304.f4.pdf]
